# Mechanism of Shenyuan Yiqi Huoxue capsule alleviating coronary microvascular dysfunction: network analysis and experimental evidence

**DOI:** 10.3389/fphar.2025.1534967

**Published:** 2025-08-04

**Authors:** Jiaping Chen, Huiwen Zhou, Hongxu Liu, Xiang Li, Huiqi Zong, Shuwen Zhang, Yunze Li, Yuxin Shi

**Affiliations:** ^1^ Department of Cardiology, Beijing Hospital of Traditional Chinese Medicine Affiliated to Capital Medical University, Beijing, China; ^2^ Department of Cardiology, Hangzhou Lin’ping District Hospital of Traditional Chinese Medicine, Zhejiang, China; ^3^ Department of Traditional Chinese Medicine, People’s Hospital of Lhasa Xizang, Lhasa, China

**Keywords:** coronary microvascular dysfunction, apoptosis, Shenyuan Yiqi Huoxue capsule, Hippo pathway, miR-302a-3p, angiogenic factors

## Abstract

**Background:**

Shenyuan Yiqi Huoxue Capsule (SYYQ) has clinical evidence to improve coronary microvascular dysfunction (CMD) by tonifying qi and removing blood stasis, but the underlying mechanism remains unclear.

**Objective:**

This study aims to explore the mechanism by which SYYQ alleviates CMD through a combination of network analysis and both *in vivo* and *in vitro* experiments.

**Methods:**

First, network pharmacology was employed to predict the mechanism of SYYQ on CMD. Building upon the findings of network pharmacology, we conducted *in vivo* experiment to verify the improvement mechanism of SYYQ in CMD rats using echocardiography, histopathology, serum biochemistry, TUNEL staining, and transmission electron microscopy (TEM). In the context of cell experiments, we evaluated the characteristic changes in mice cardiac microvascular endothelial cells (MCMECs) and the molecular mechanism of SYYQ through cell transfection, TEM, Western blotting, and qRT-PCR.

**Results:**

The results of network pharmacology suggest that SYYQ may enhance CMD through pathways related to apoptosis and vascular growth. Animal experiments demonstrated that SYYQ alleviated apoptosis, promoted microvascular opening, and reduced myocardial injury in CMD rats. Furthermore, cell experiments indicated that SYYQ mitigated apoptosis in hypoxic (Hyp) MCMECs, promoted the production of angiogenic factors. Furthermore, downregulation of miR-302-3p levels and activation of Hippo pathway were observed in Hyp MCMECs, which can be inhibited by SYYQ. When miR-302a-3p was overexpressed or the Hippo pathway was inhibited, the efficacy of SYYQ in promoting the production of angiogenic factors and inhibiting apoptosis in Hyp MCMECs was significantly enhanced. Additional studies revealed that miR-302a-3p negatively regulated LATS2.

**Conclusion:**

SYYQ improves CMD by promoting the production of angiogenic factors and inhibiting apoptosis via miR-302a-3p/Hippo.

## 1 Introduction

Coronary microvascular dysfunction (CMD) is a clinical syndrome characterized by structural and/or functional abnormalities of coronary microvessels due to pathogenic factors (Zhang Y. L. et al., 2017), which is closely associated with poor prognosis in patients with ischemic heart disease (IHD) (Bairey et al., 2020). Research has demonstrated that apoptosis of microvascular endothelial cells, induced by microvascular embolism, inflammation, and other factors, leads to endothelial dysfunction, serving as a critical pathophysiological basis for the onset and progression of CMD(Deng, 2021). The proliferation of microvascular endothelial cells following injury plays a vital role in the recovery of coronary microvascular (Kruger-Genge et al., 2019). The *Evaluation and Treatment of Coronary Microvascular Dysfunction* by the European Society of Cardiology notes that “There are no therapeutic strategies focused on specifically treating CMD and the microvasculature. There is an urgent need to identify novel and specific targets for therapy” (Padro et al., 2020). This highlights the potential for effective intervention using traditional Chinese medicine (TCM).

Shenyuan Yiqi Huoxue Capsule (SYYQ) is a Chinese herbal compound preparation that has been shown to offer various myocardial protective effects in patients with IHD. These effects include improving glucose and lipid metabolism disorders, protecting vascular endothelial function, alleviating myocardial ischemia and ischemia-reperfusion injury, and providing myocardial protection during percutaneous coronary interventions (PCI) (Chou et al., 2016; Shang et al., 2017; Tong et al., 2016; Xia, 1996; Zhang Y. et al., 2017; Zhou et al., 2017; Zhou M. et al., 2019; Zhu et al., 2017). Notably, alleviating apoptosis is one of its key mechanisms of function. Recent study utilizing the coronary index of microcirculatory resistance (IMR) based on invasive pressure wire technology found that SYYQ significantly improved coronary microvascular function during perioperative PCI(Li, 2020). Further elucidation of its mechanisms will be essential for objectively evaluating the improvement of CMD with SYYQ.

The Hippo pathway is crucial for regulating cell proliferation and organ size (Liu and Martin, 2019). Most previous studies on the Hippo pathway have primarily focused on tumor biology, where it promotes the proliferation of tumor cells (Fu et al., 2022). Recently, differential gene analysis and co-expression network analysis revealed that the Hippo pathway is notably involved in patients with microvascular dysfunction in acute coronary syndrome compared to those with normal coronary artery flow (Dai, 2022). Tian et al. (Tian et al., 2015) demonstrated that miR-302a-3p could inhibit the Hippo signaling pathway, which in turn promotes the regeneration and repair of adult cardiomyocytes following myocardial infarction. Additionally, accumulating evidence indicates that Hippo pathway plays a significant role in regulating vascular endothelial functions, including oxidative stress, inflammation, angiogenesis, and apoptosis (Zhang et al., 2022). However, no studies have reported on the Hippo pathway’s regulation of coronary microvascular endothelial cells (CMECs) damage in the context of CMD. In summary, in light of the clinical evidence that SYYQ improves CMD, we propose a scientific hypothesis that SYYQ may ameliorate CMD through the Hippo signaling pathway mediated by miR-302a-3p, thus exerting a myocardial protective effect.

**FIGURE 1:**
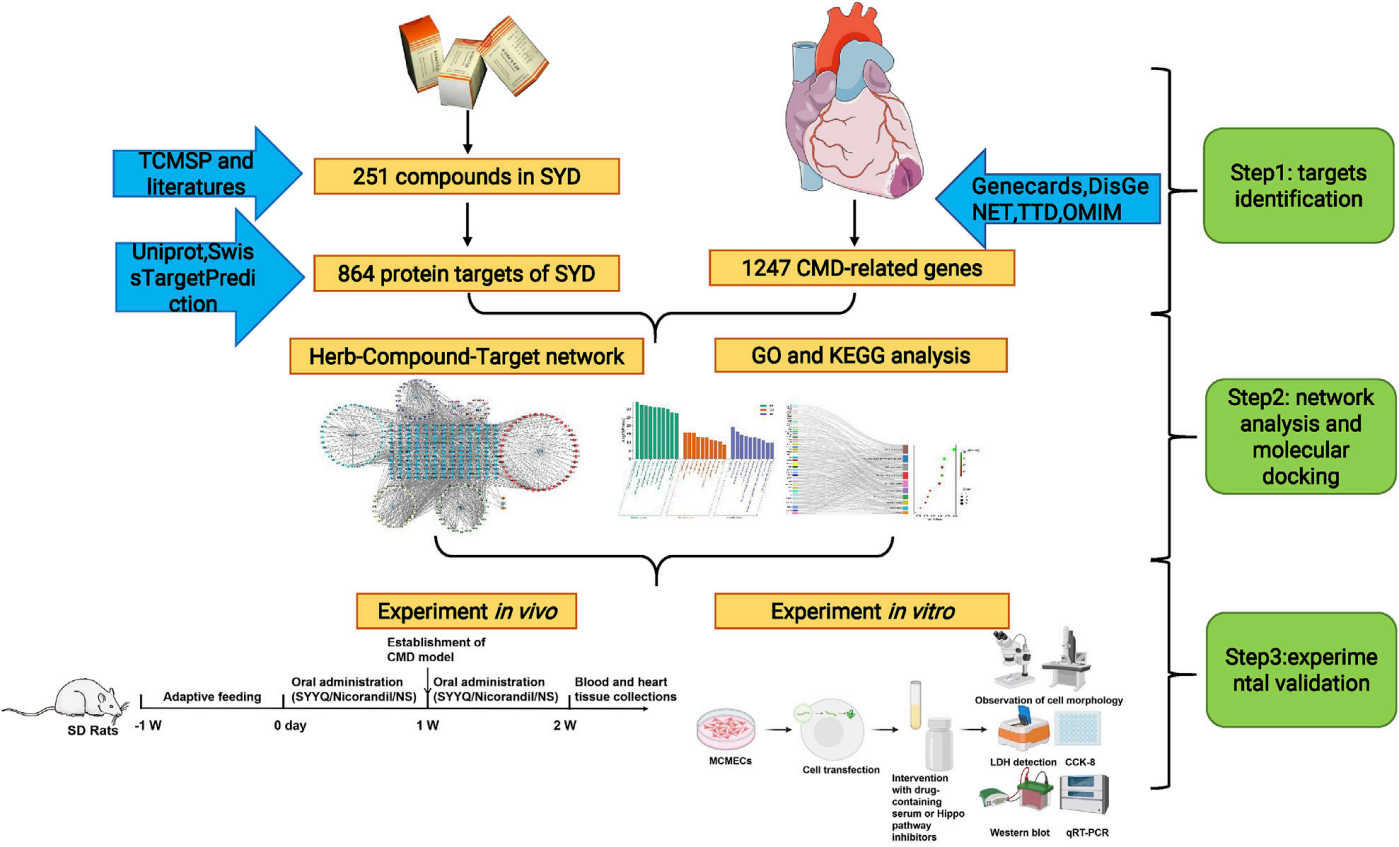
The flowchart of the experimental procedure.

TCM compounds are characterized by their multi-component nature, complexity, and ability for coordinated integration (Zhang et al., 2019). Network pharmacology, which operates at the system level and within biological networks, has become a valuable approach in studying the complex systems of TCM(An et al., 2021). Therefore, this study employs network pharmacology alongside *in vivo* and *in vitro* experiments to explore the mechanisms by which SYYQ improves CMD (see Figure 1).

## 2 Materials and methods

### 2.1 Preparation and quality control of the SYYQ

SYYQ is provided by the Traditional Chinese Medicine Preparation Department of Beijing Hospital of Traditional Chinese Medicine, with production batch number Z20053327. It consists of Astragalus mongholicus (*Astragalus mongholicus* Bunge), Hirudo (*Hirudo* nipponica Whitman), Codonopsis pilosula (*Codonopsis pilosula* (Franch.) Nannf.), Eupolyphaga Seu Steleophaga (*Eupolyphaga* sinensis Walker), Pheretima (*Pheretima* aspergillum (E.Perrier)), Scrophularia ningpoensis (*Scrophularia ningpoensis* Hemsl.), Salvia miltiorrhiza (*Salvia miltiorrhiza* Bunge), Corydalis yanhusuo (*Corydalis yanhusuo* (Y.H.Chou & Chun C. Hsu) W.T.Wang ex Z.Y.Su & C.Y.Wu) (Table 1). The preparation method of SYYQ is described in detail in Supplementary Table S7. The pharmacodynamic data of SYYQ is described in Supplementary Material 2. The recommended daily intake for humans is 12 capsules, with each capsule containing 760 mg of raw medicinal ingredients. Previous toxicity studies have demonstrated that the maximum daily dose of SYYQ can reach 412.9 times the clinical dosage (Supplementary Material 1). Given that our research group has mainly focused on acute myocardial infarction (Chu et al., 2016) and ischemia/reperfusion (Liu et al., 2013) rat models in the past, this experiment represents the first application of SYYQ in a CMD model. Based on the absence of significant coronary artery thrombosis and the lack of severe heart failure of CMD rat, and taking into account the daily gavage dose in rat, we set the dosage of the low, medium, and high dose groups to 1.5, 3, and 6 times the clinical dose, respectively. According to the body surface area ratio (Reagan-Shaw et al., 2008), the SYYQ low-dose group was set at 1.5 g/kg/day, the medium-dose group at 3 g/kg/day, and the high-dose group at 6 g/kg/day. The SYYQ were dissolved in saline and set the concentration to 1 mg/mL. The quality control of SYYQ has been published in previous studies (Lou, 2021) and described in Supplementary Material 2.

**TABLE 1:** Eight botanical drugs of SYYQ.

Chinese name	Name of publishing	Used parts	Weight (g)
Huangqi	*Astragalus mongholicus* Bunge [Fabaceae; *Astragali Radix*]	Root	12
Shuizhi	*Hirudo nipponica* Whitman [hirudinidae; *Hirudo*]	All parts	3
Dangshen	*Codonopsis pilosula* (Franch.) Nannf. [Campanulaceae; C*odonopsis Radix*]	Root	10
Tubiecong	*Eupolyphaga sinensis* Walker [Corydidae; *Eupolyphaga Steleophaga*]	All parts	6
Dilong	*Pheretima aspergillum* (E.Perrier) [*Pheretima*]	All parts	5
Xuanshen	*Scrophularia ningpoensis* Hemsl. [Scrophulariaceae; S*crophulariae Radix*]	Root	5
Danshen	*Salvia miltiorrhiza* Bunge [Lamiaceae; S*alviae Miltiorrhizae Radix Et Rhizoma*]	Root and rhizome	15
Yanhusuo	*Corydalis yanhusuo* (Y.H.Chou & Chun C.Hsu) W.T.Wang ex Z.Y.Su & C.Y.Wu [Papaveraceae; *Corydalis Rhizoma*]	Tuber	5

### 2.2 Network pharmacology

We collected the components of drugs in SYYQ using the TCMSP database (https://old.tcmsp-e.com/tcmsp.php) (Ru et al., 2014), SwissTargetPrediction database (http://www.swisstargetprediction.ch/) (Daina et al., 2019), and relevant literature (Lou et al., 2021; Wang et al., 2021; Wu et al., 2023). In the TCMSP database, the active compounds were filtered by integrating the pharmacokinetic properties comprising oral bioavailability (OB) ≥30% (Walters, 2002), drug-likeness (DL) ≥0.18 (Xu et al., 2012). In the SwissTargetPrediction database, the probability value of potential target proteins ≥0.1. Based on these drug components, we obtained the associated drug targets, which were uploaded to the UniProt platform (https://www.uniprot.org/) (Consortium, 2023) for unified conversion into Gene Symbols. Subsequently, we searched for disease targets in the GeneCards (https://www.genecards.org/) (Stelzer et al., 2016), DisGeNET (https://www.disgenet.org/) (Pinero et al., 2021), TTD (http://db.idrblab.net/ttd) (Zhou et al., 2022), and OMIM databases (https://www.omim.org/) (Li et al., 2014) using search terms such as “coronary microvascular disease”, “coronary microvascular dysfunction” or “microvascular angina” and specifying “*H. sapiens*” as the species filter. We utilized Venn diagrams to identify the intersection targets of diseases and drugs, which were then imported into Cytoscape 3.7.1 software to construct a “drug-component-intersection target” network. Finally, we performed protein-protein interaction (PPI) network analysis, gene ontology (GO) enrichment, and Kyoto Encyclopedia of Genes and Genomes (KEGG) pathway analysis using the STRING online platform (https://string-db.org/) (Szklarczyk et al., 2021) and the Metascape database (https://metascape.org/gp/index.html) (Zhou M. et al., 2019).

### 2.3 Chemicals, kits, and reagents

Nicorandil tablets (H61022860) were purchased from Hanfeng Pharmaceutical (Xi’an, China). Sodium Laurate (S30244-250g) was sourced from Yuanye Bio Technology (Shanghai, China). Heidenhain staining solution (DH0013) was obtained from Leadene Biotechnology (Beijing, China). Hematoxylin staining solution (MD911477), eosin (MD911467), the enzyme-linked immunosorbent assay (ELISA) kit for cardiac troponin I (cTnI) (MD121047), creatine kinase isoenzyme (CK-MB) ELISA kit (MD121048), Caspase-9 ELISA kit (MD121046), Bcl-2 ELISA kit (MD120042), vascular endothelial growth factor (VEGF) ELISA kit (MD120404), fibroblast growth factor (FGF) ELISA kit (MD121049), and bicinchoninic acid (BCA) protein detection kit (MD913053) were purchased from MDL Biomedical Technology (Beijing, China). Fetal bovine serum (FBS) (SH30070.03) was procured from Hyclone (USA), and the endothelial cell growth supplement (ECGS) (1052) was obtained from ScienCell (USA). The lactate dehydrogenase (LDH) kit (A020-2) was purchased from Jiancheng Bioengineering (Nanjing, China). The Cell Counting Kit-8 (CCK-8) reagent (DCM21822) was acquired from Fluorescence (Beijing, China). The TdT-mediated dUTP Nick-End Labeling (TUNEL) kit (C1088) was sourced from Beyotime Biotechnology (Shanghai, China). Trizol reagent (10296028) and the SuperScript III RT reverse transcription kit (11752050) were purchased from Invitrogen (Waltham, USA). Actin (Ab7817) antibodies were acquired from Abcam (Cambridge, UK). Antibodies for LATS1 (AF7669), LATS2 (DF7516), Mob1 (DF6417), YAP (AF6328), p-YAP (AF3328), caspase-9 (AF5240), Bcl-2 (AF6139), VEGF (AF5131), and FGF2 (DF6038) were purchased from Affinity Biosciences (Jiangsu, China).

### 2.4 Animals

Sixty healthy adult male Sprague Dawley (SD) rats (weight: 200 ± 20g) were obtained from Beijing Huafukang Biotechnology Co., Ltd. (certificate number: SCXK (Beijing) 2019-0008) for *in vivo* experiments. Additionally, 180 healthy adult male C57BL/6J mice (weight: 20–25 g) were acquired from Speyford (Beijing) Biotechnology Co., Ltd. (certificate number: SCXK (Beijing) 2019-0010) for *in vitro* experimental preparation of drug-containing serum. The animals were adaptively raised under standard conditions for 1 week with free access to a standard diet and distilled water. This study was approved by the Experimental Animal Ethics Committee of Kangtai Medical Laboratory Services Hebei Co., Ltd. (MOL2022070705), and all animal operations were conducted following the Guide for the Care and Use of Laboratory Animals published by the US National Institutes of Health (publication, No. 85-23, 2011).

### 2.5 Preparation of drug-containing serum

We prepared control serum and serum containing SYYQ in advance. C57BL/6J mice were randomly divided into two groups: the treatment group (n = 140) received 0.05 g/kg SYYQ by gavage, while the control group (n = 40) was given an equal volume of saline twice daily for five consecutive days. Blood was collected from the eye socket 1–2 h after the last administration, allowed to stand for 2 h, centrifuged at 3,000 rpm for 10 min, and then collected. The serum was inactivated at 56°C and stored at −20°C. Based on previous research, 5% medicated serum was selected for use in the intervention treatment (Liu et al., 2013). Prior to cell experiments, both SYYQ and control sera were diluted to 5% with Dulbecco’s Modified Eagle’s Medium (DMEM)/F12 culture medium.

### 2.6 Animal grouping and CMD model preparation

SD rats were randomly assigned to six groups (10 rats per group): the sham surgery group, the CMD group, the SYYQ low dose group (SYYQ-L, 1.5 g/kg/d), the SYYQ medium dose group (SYYQ-M, 3 g/kg/d), the SYYQ high dose group (SYYQ-H, 6 g/kg/d), and the nicorandil group (2.7 mg/kg/d). The SYYQ-L, SYYQ-M, SYYQ-H, and nicorandil groups received their respective oral doses of SYYQ and nicorandil for 7 days before and after modeling, while the sham surgery and CMD groups were orally administered normal saline. Nicorandil serves as a clinical treatment for CMD and is often used as a positive control drug (Liu H. et al., 2021).

After 7 days of administration, the model was established using intraperitoneal anesthesia with 2% pentobarbital sodium in the rats. Invasive endotracheal intubation was performed for respiratory support, followed by a longitudinal incision between the third and fourth ribs of the left anterior chest. The pericardium was excised to fully expose the aortic root. After clamping the aorta, 200 µg of sodium laurate (1 g/mL) was injected into the left ventricle. The rats in the sham surgery group underwent the same surgical procedure, but were injected with 0.2 mL of physiological saline instead. Following the injection, the chest gas was expelled, and the incision was quickly sutured. Successful modeling was confirmed by hematoxylin-eosin (HE) staining, which revealed inflammatory cell infiltration in the myocardial infarction area, the formation of coronary microvascular thrombosis, and the dissolution and rupture of myocardial cells (Cui et al., 2023).

### 2.7 Cell culture and treatment

The mice coronary microvascular endothelial cells (MCMECs) was obtained from Wuhan Ponosi Life Technology Co., Ltd. The cells were cultured in DMEM supplemented with 20% FBS, 1% ECGS, and 100 μg/mL penicillin/streptomycin at 37°C in an atmosphere of 95% air and 5% CO_2_ for 24 h. Subsequently, MCMECs were washed and cultured in low glucose serum-free DMEM in a 1% air, 94% N_2_, and 5% CO_2_ incubator for 12 h to establish a model of hypoxia-induced injury. Prior to hypoxia, control serum (5%), SYYQ-containing serum (5%), and a pathway blocker (TDI-011536, 3 µM) were added to the corresponding groups of cells. The culturing principle is detailed in the Supplementary Material 3.

Following the manufacturer’s instructions, Hieff Trans™ Liposomal Transfection Reagent (Yeasen) was employed for the transfection of miR-302a-3p mimic and miR-302a-3p inhibitor into the cells. The cells were divided into the following groups: (1) blank control group (Ctrl); (2) hypoxia group (Hyp): MCMECs were cultivated in a 1% air- 94% N_2_-5% CO_2_ environment for 12 h; (3) blank serum + hypoxia group (Blank serum + Hyp): MCMECs were cultured in a hypoxic environment using blank serum DMEM for 12 h; (4) SYYQ + hypoxia group (Hyp + SYYQ): MCMECs were cultured in a hypoxic environment using SYYQ-containing serum DMEM for 12 h; (5) Hippo pathway inhibitor + hypoxia group (Hyp + TDI-011536): MCMECs were cultured in DMEM containing the TDI-011536 for 12 h in a hypoxic environment; (6) miR-302a-3p mimic + hypoxia group (Hyp + miR-302a-3p mimic): MCMECs were transfected with miR-302a-3p mimic and subsequently cultured in a hypoxic environment for 12 h; (7) miR-302a-3p mimic + SYYQ + hypoxia group (Hyp + miR-302a-3p mimic + SYYQ): MCMECs were transfected with miR-302a-3p mimic and then incubated in SYYQ-containing serum DMEM in a hypoxic environment for 12 h; (8) miR-302a-3p inhibitor + hypoxia group (Hyp + miR-302a-3p inhibitor): MCMECs were transfected with miR-302a-3p inhibitor and then cultured in a hypoxic environment for 12 h; (9) miR-302a-3p inhibitor + SYYQ + hypoxia group (Hyp + miR-302a-3p inhibitor + SYYQ): MCMECs were transfected with miR-302a-3p inhibitor and then incubated in SYYQ-containing serum DMEM in a hypoxic environment for 12 h.

### 2.8 Echocardiography

Echocardiography was performed on the rats using Vevo 1100 (VisualSonics, Bothwell,WA,USA) 7 days after surgery. All echocardiographic measurements (left ventricular ejection fraction (LVEF), left ventricular braxial shortening rate (LVFS), end-systolic left ventricular diameter (LVIDs), and end-diastolic left ventricular diameter (LVIDd)) in each group were measured by professional technicians.

### 2.9 Histopathological examination

Following the assessment of cardiac function, the rats were euthanized. The ventricular tissue was fixed in 4% paraformaldehyde, dehydrated, embedded in paraffin, and sectioned into 4 μm thick slices. According to the manufacturer’s instructions, the microvascular diameter, opening rate, and occlusion rate were evaluated using HE staining, while the size of myocardial microinfarctions was determined using Heidenhain staining. The images were captured by an intelligent tissue slice imaging analysis system and analyzed with Image-Pro Plus 6.0 software (Media Cybernetics, Rockville, MD, USA).

### 2.10 Detection of serum cTnI, CK-MB, caspase-9, Bcl-2, VEGF, and FGF

Blood samples were obtained from the posterior orbital venous plexus of rats prior to modeling and at 4 h, 24 h, and 7 days post-modeling. Serum levels of cTnI, CK-MB, caspase-9, Bcl-2, VEGF, and FGF were quantified using an ELISA kit in accordance with the manufacturer’s instructions.

### 2.11 Transmission electron microscopy (TEM)

Cardiac tissue and MCMECs were imaged by TEM to observe the cellular structure of cardiomyocytes and microvascular endothelial cells. Myocardial tissue samples and MCMECs were fixed with glutaraldehyde, then cleaned, dehydrated, embedded, sliced, and stained. Ultrathin sections were observed using TEM (Japan, JEOL, JEM1230).

### 2.12 LDH detection

Cell damage was assessed by measuring LDH levels using an LDH assay kit according to the manufacturer’s instructions.

### 2.13 Cell viability detection

Cell viability was assessed using the CCK-8 kit according to the manufacturer’s instructions.

### 2.14 Detection of apoptosis

According to the manufacturer’s instructions, TUNEL staining was used to evaluate apoptosis in rat myocardial tissue and MCMECs cells.

### 2.15 Quantitative real-time polymerase chain reaction (qRT-PCR)

Total RNA was extracted from MCMECs using Trizol reagent (Invitrogen, Waltham, USA) according to the manufacturer’s instructions. cDNA was synthesized using reverse transcription kit (ABI-invitrogen, Waltham, USA). Target genes were quantified by SYBR Green PCR premix and detected by Applied biosystems (Thermo Fisher, USA). The target primers were miR-302a-3p, Caspase-9, Bcl-2, VEGF, FGF2, LATS1, LATS2, Mob1 and YAP. Actin serves as an internal control gene. Gene levels were measured by 2^−ΔΔCt^ relative quantification. The primers used in this study are shown in Table 2.

**TABLE 2:** Primer sequences.

Target Name	Primer	
Actin	F	CTC​CTG​AGC​GCA​AGT​ACT​CT
	R	TAC​TCC​TGC​TTG​CTG​ATC​CAC
LATS1	F	CTT​CAC​CTA​TCA​CTG​TTC​GGA
	R	ATG​AGA​CTT​CAG​GAC​GTT​C
LATS2	F	AAA​TCA​AGA​CTC​TAG​GCA​TCG​GT
	R	GCC​ACT​TGA​TTC​CGG​TTC​AGG
Mob1	F	AAA​GCC​AAT​TAA​GTG​TTC​TGC
	R	CGC​TTC​AGA​ATA​GTC​TTT​GCC
YAP	F	ACC​ATC​AGC​CAA​AGC​ACC​CT
	R	TGC​CAA​GGT​CCA​CAT​TTG​TCC​CA
Caspase-9	F	GAA​GAA​CGA​CCT​GAC​TGC​CAA
	R	GAC​CAC​CAC​AAA​GCA​GTC​CA
Bcl-2	F	ACT​TCT​CTC​GTC​GCT​ACC​GTC
	R	CCC​CAT​CCC​TGA​AGA​GTT​CCT
VEGF	F	GCA​GAC​CTC​CCC​ACC​ATC​CCT
	R	ACT​GGC​CGG​AGC​ACA​CCA​AC
FGF2	F	GCG​ACC​CAC​ACG​TCA​AAC​TA
	R	CCG​TCC​ATC​TTC​CTT​CAT​AGC

### 2.16 Western blot (WB) analysis

After the complete cleavage of MCMECs in each group, the protein was extracted, and the protein concentration was determined by BCA method. It was then separated by sodium dodecyl sulphate-polyacrylamide gel electrophoresis (SDS-PAGE) and transferred to a polyvinylidene fluoride (PVDF) membrane (Millipore, ISEQ00010). The membranes were blocked with PBS containing 5% skim milk at room temperature for 1 h and then incubated with the corresponding primary antibodies (Bcl-2, VEGF, FGF2, LATS1, LATS2, Mob1, YAP, p-YAP, and Actin) at 4°C (1:1000) overnight. After washing, they were incubated with the corresponding secondary antibody (1:300) at room temperature for 1 h. Chemiluminescence imaging system was used for imaging. The intensity of protein bands was quantified by ImageJ software.

### 2.17 Statistical analysis

If the data follows a normal distribution and the variances across groups are homogeneous, a t-test or one-way analysis of variance (ANOVA) was used for statistical analysis by using GraphPad Prism 8.0 (GraphPad Software Inc., CA, USA), and the results were presented as the mean ± standard deviation ( $\bar{\chi }$ ± s). If the data is non-normally distributed or the variances are heterogeneous, non-parametric method such as the rank-sum test was used for analysis by using GraphPad Prism 8.0 (GraphPad Software Inc., CA, USA), and the results were expressed as median and interquartile range [M (QL, QU)]. Some FGF indicators in the *in vivo* experiment were tested using rank-sum test, while the remaining data were analyzed using t-test and ANOVA. When *P* < 0.05, the difference was considered statistically significant.

## 3 Result

### 3.1 Results of a network pharmacology study of SYYQ for the treatment of CMD

**FIGURE 2:**
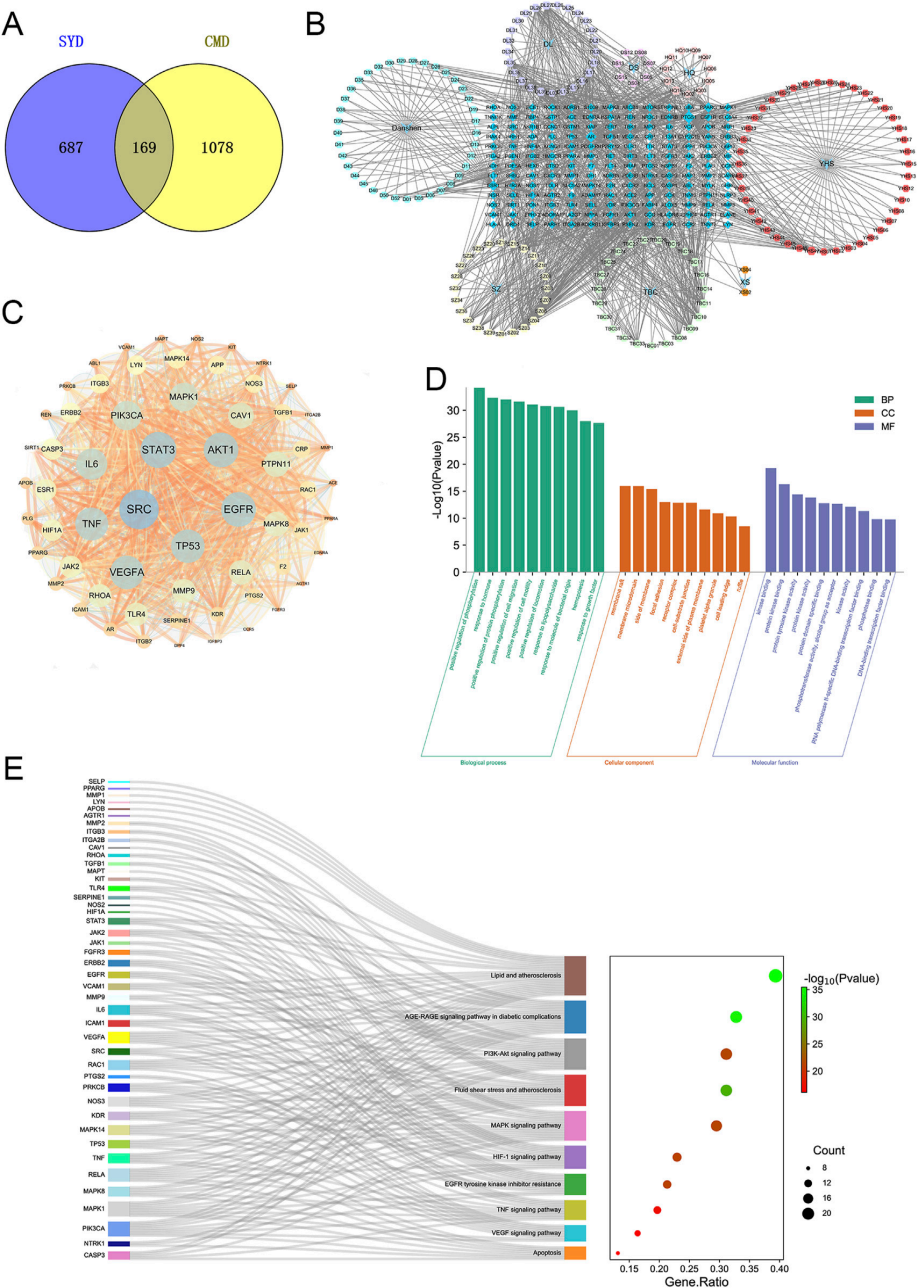
Network pharmacology analysis of the SYYQ improving CMD. **(A)** intersection target of SYYQ and CMD; **(B)** “Drug - component - intersection target” network; **(C)** Protein-protein interaction network; **(D)** Biological processes, cell components and molecular functions identified in the enrichment analysis of 61 key target genes; **(E)** GO and KEGG enrichment analyses of 61 key targets.

Through the TCMSP, SwissTargetPrediction, and Uniprot databases, we identified 251 potential compounds in SYYQ (Supplementary Table S1), corresponding to 864 targets (Supplementary Table S2). In this study, the chemical composition of SYYQ was analyzed using ultra-performance liquid chromatography coupled with quadrupole-time-of-flight mass spectrometry (see Supplementary Material 2). However, animal-derived drug components in SYYQ could not be identified due to limitations in current detection techniques. Therefore, potential active compounds obtained through online pharmacological analysis were used in subsequent target prediction. A total of 1247 disease targets were extracted from the GeneCards, DisGeNET, TTD, and OMIM databases (Supplementary Table S3). As illustrated in Figure 2A, there were 169 intersection targets identified between SYYQ and CMD (Supplementary Table S4). After importing the intersection targets, drugs, and ingredients into Cytoscape 3.7.1, we constructed a “drug-ingredient-intersection target” network (Figure 2B). The intersection targets were subsequently imported into the STRING database, and network topology analysis was conducted using Cytoscape 3.7.1. 61 key targets with topological feature values (freedom, interiority, and compactness) above the median were selected (Figure 2C; Supplementary Table S5).

61 key targets were imported into Metascape database for GO and KEGG enrichment analysis. We took the top ten GO entries with *P* < 0.01 and plotted a bar graph (Figure 2D; Supplementary Table S6). GO enrichment analysis showed that the biological process was mainly enriched in positive regulation of phosphorylation, response to hormone, etc. Most targets targeted cellular components such as membrane rafts, membrane microdomains. They were involved in molecular functions including kinase binding, protein kinase binding, and protein tyrosine kinase activity. The KEGG enrichment results revealed that the key targets were mainly enriched in lipid metabolism, atherosclerosis, the AGE-RAGE signaling pathway in diabetic complications, the TNF signaling pathway, the VEGF signaling pathway, apoptosis, and other signaling pathways (Figure 2E).

Combined with the above network pharmacological results, it can be concluded that SYYQ may improve coronary microvascular through the pathways related to apoptosis and vascular growth.

### 3.2 SYYQ improves cardiac function and alleviates myocardial injury in CMD rats

**FIGURE 3:**
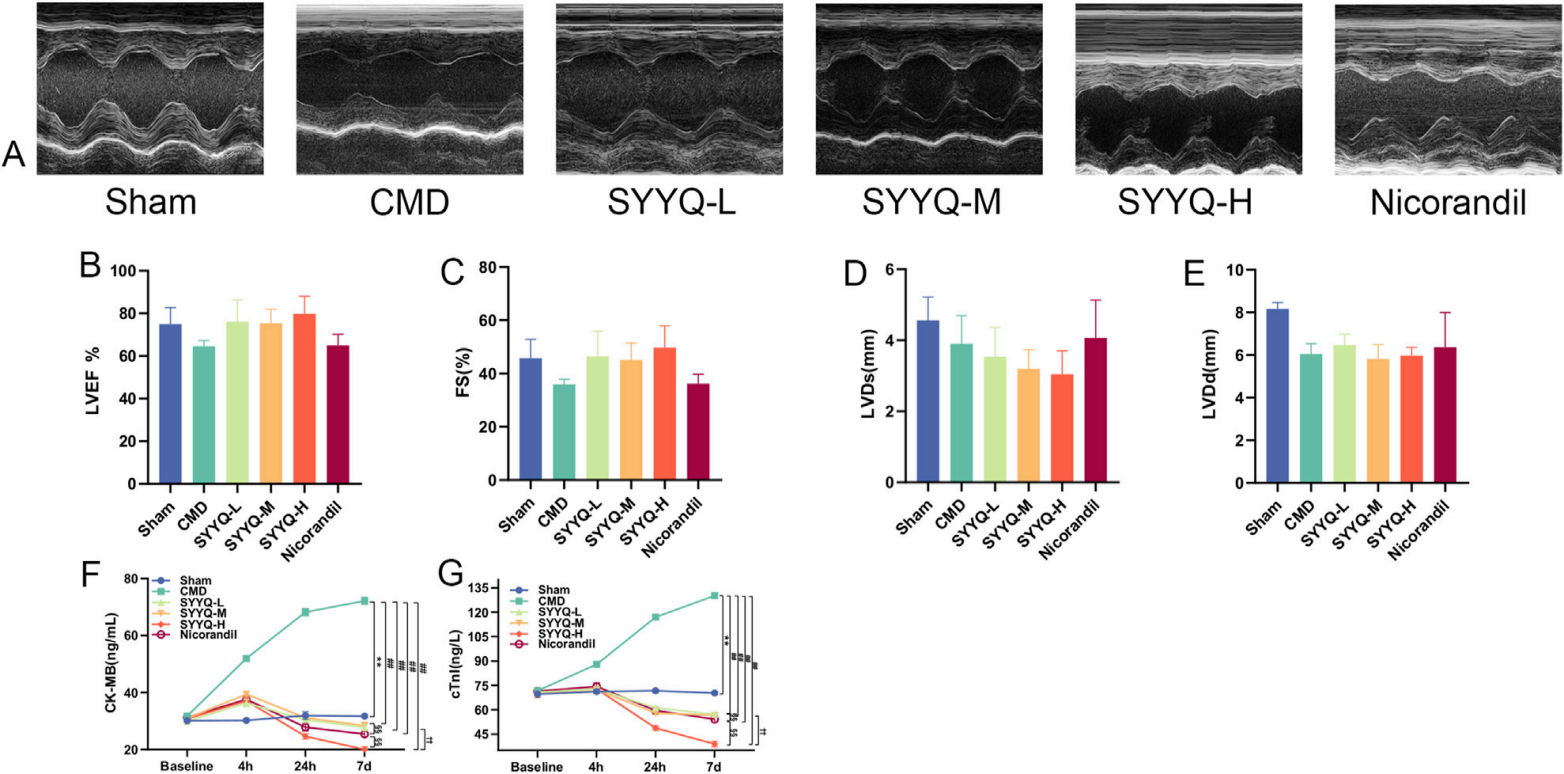
SYYQ improves cardiac function and alleviates myocardial injury in CMD rats. Note: **(A)** Representative echocardiographic images of rats in each group; **(B–E)** LVEF, FS, LVID(s), LVID(d) for each group (n = 10); **(F–G)** Serum levels of CK-MB and cTnI in each group (n = 10). ^**^ *P* < 0.01 vs. Sham; ^##^ *P* < 0.01 vs. CMD; ^§§^ *P* < 0.01 vs. Nicorandil; ^†^^†^ *P* < 0.01 vs. SYYQ-L.

In terms of echocardiography, we observed that LVEF, FS, LVID(s) and LVID(d) of rats in CMD group showed a decrease trend, and after SYYQ intervention, LVEF and FS showed a recovery trend. The above differences were not statistically significant, potentially due to an insufficient sample size (*P* > 0.05) (Figures 3A–E; Supplementary Table S8). Compared with Sham group, the levels of serum cTnI and CK-MB in CMD group were increased (*P* < 0.05). After SYYQ intervention, cTnI and CK-MB levels showed a reduction to some extent, with the high dose of SYYQ demonstrating a greater improvement than the low dose (Figures 3F,G; Supplementary Tables S9, S10).

### 3.3 SYYQ improves the pathomorphology of CMD rats and hyp MCMECs

**FIGURE 4:**
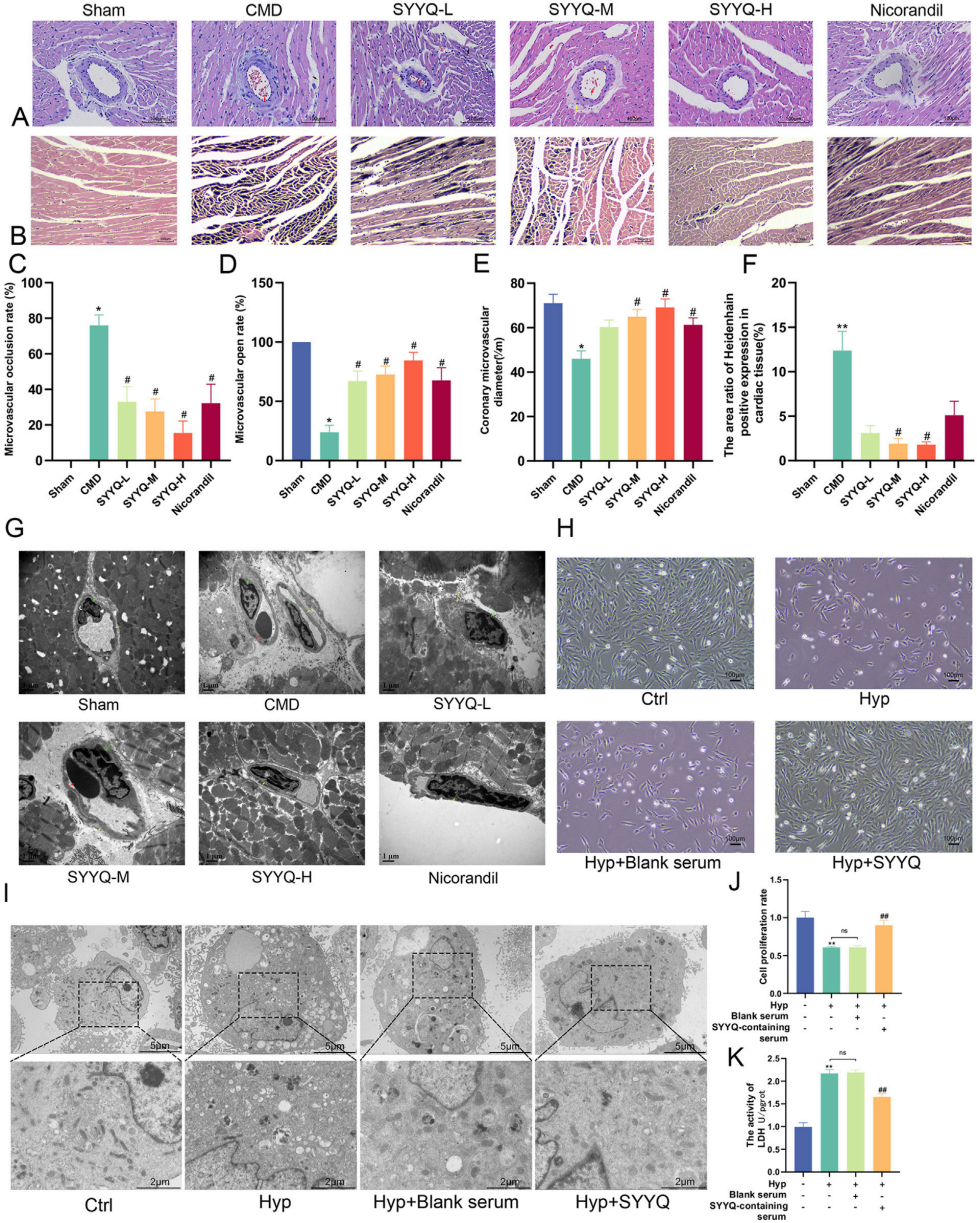
SYYQ improves the pathomorphology of CMD rats and hyp MCMECs. Note: **(A)** HE staining of rat heart tissue; **(B)** Heidenhain staining of rat heart tissue. **(C–E)** Occlusion, opening and diameter of coronary microvessel in rats (n = 6); **(F)** Statistical results of microinfarct size in rats (n = 6); **(G)** Micromorphology of ischemic myocardium in rats; **(H)** Optical microscope images of MCMECs morphological changes; **(I)** Transmission electron microscope images of MCMECs; **(J)** Cell viability assay; **(K)** Cell damage assay. ^**^ *P* < 0.01 vs. Sham *in vivo* or Ctrl *in vitro*; ^##/#^ *P* < 0.01/0.05 vs. CMD *in vivo* or Hyp *in vitro.*

HE staining revealed that CMD induced inflammatory infiltration in myocardial tissue, accompanied by intercellular substance edema, myocardial dissolution, rupture, and severe microvascular cavity embolism, indicating successful modeling (Cui et al., 2023). SYYQ intervention mitigated myocardial inflammatory infiltration, rupture, and microvascular thrombosis (Figure 4A). In CMD group, coronary microvessel thrombosis was serious, the number of open microvessels was reduced, and the diameter of microvessels was narrowed, and the above conditions could be improved after the intervention of different doses of SYYQ and nicorandil (*P* < 0.05) (Figures 4C–E; Supplementary Tables S11, S13). Heidenhain staining showed that both medium and high doses of SYYQ effectively reduced the myocardial area of ischemic black stain (*P* < 0.05, Figures 4B,F; Supplementary Table S14). TEM results of rat heart tissue showed that medium and high doses of SYYQ could reduce the morphological disorder of cardiomyocytes, the swelling of CMECs nucleus and the thrombosis of vascular lumen (Figure 4G). Additionally, in Hyp MCMECs, TEM observations revealed that SYYQ intervention could ameliorate Hyp-induced apoptosis (characterized by chromatin concentration and margination), mitochondrial cristae swelling or disappearance, and cytoplasmic vacuolar degeneration (Figures 4H,I), whilst also enhancing cell viability (Figure 4J) and reducing cell damage (Figure 4K). Together, these findings suggest that SYYQ has a cardio-protective effect by alleviating CMD-associated endothelial cell damage.

### 3.4 SYYQ promotes the generation of angiogenic factors and inhibits apoptosis in CMD rats and hyp MCMECs

**FIGURE 5:**
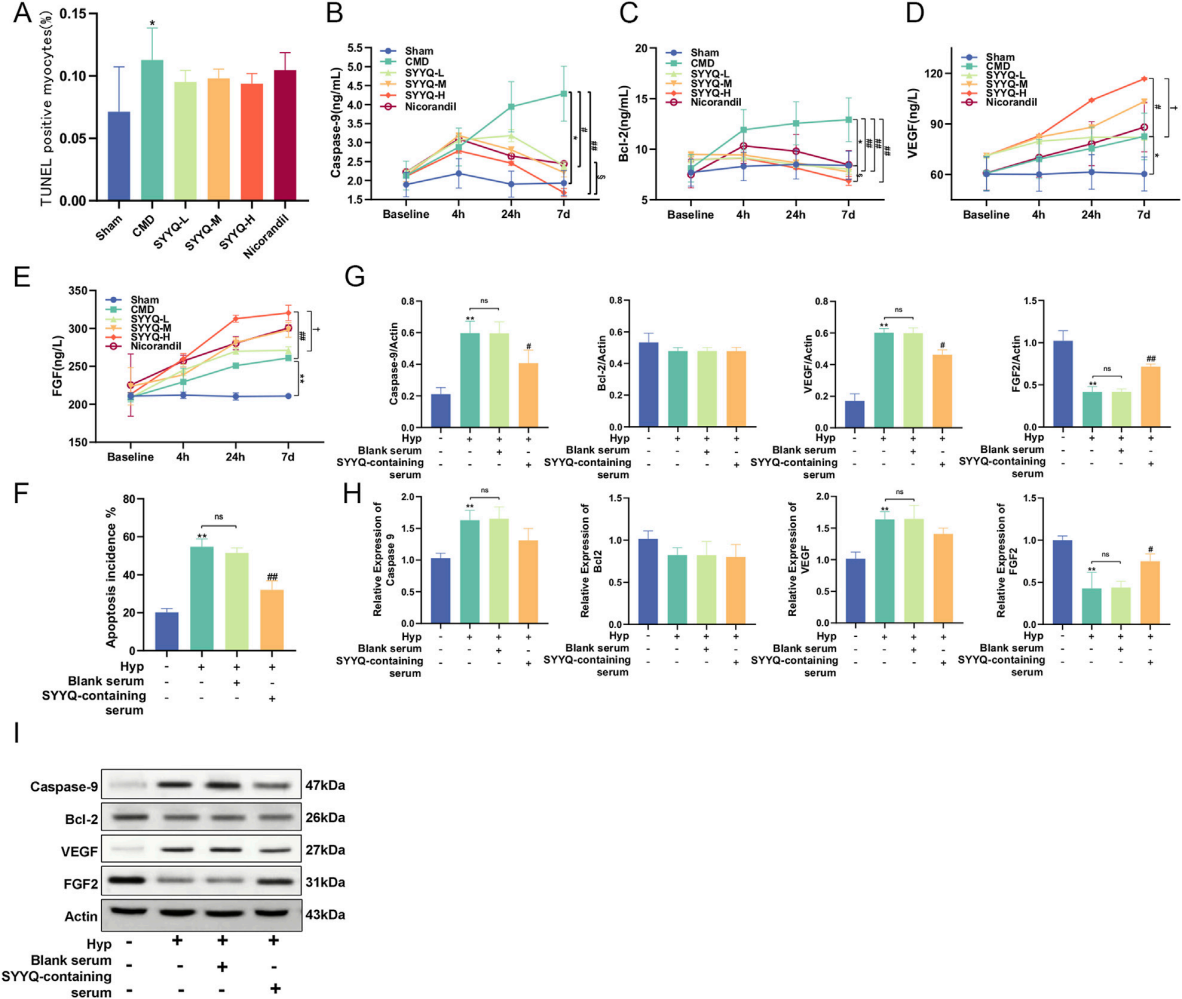
SYYQ promotes the generation of angiogenic factors and inhibits apoptosis in CMD rats and Hyp MCMECs. Note: **(A)** TUNEL assay was used to determine the apoptosis rate *in vivo* (n = 6); **(B–E)** ELISA results of Caspase-9, Bcl-2, VEGF and FGF *in vivo* (n = 10); **(F)** The apoptosis rate was measured *in vitro* by TUNEL assay, n = 3. **(G,I)** The expressions of Caspase-9, Bcl-2, VEGF and FGF2 were detected by Western Blot. **(H)** mRNA expression levels of Caspase-9, Bcl-2, VEGF and FGF2 were detected by qRT-PCR. n = 3. ^**/*^ *P* < 0.01/0.05 vs. Sham *in vivo* or Ctrl *in vitro*; ^##/#^ *P* < 0.01/0.05 vs. CMD *in vivo* or Hyp *in vitro*; ^§^ *P* < 0.05 vs. Nicorandil *in vivo*; ^†^ *P* < 0.05 vs. SYYQ-L *in vivo.*

TUNEL staining was used to evaluate the apoptosis rate of cardiomyocytes in rats. Apoptotic cells increased in CMD group (*P* < 0.05), and SYYQ intervention exhibited a trend towards reducing cardiomyocyte apoptosis, although this difference was not statistically significant (*P* > 0.05) (Figure 5A; Supplementary Table S15; Supplementary Figure SF1). ELISA results indicated that Caspase-9 and Bcl-2 levels were heightened in the CMD group, but these changes were reversed by SYYQ treatment, while VEGF and FGF levels were significantly increased due to SYYQ treatment, and all of the aforementioned effects are intensified with prolonged drug intervention (Figures 5B–E; Supplementary Tables S16-S19). In Hyp MCMECs, it was also observed that SYYQ intervention significantly reversed apoptosis (*P* < 0.05) by TUNEL staining (Figure 5F; Supplementary Figure F2). WB and qRT-PCR results showed that the expressions of Caspase-9 and VEGF in Hyp MCMECs were significantly increased (*P* < 0.05), and the expression of FGF2 was significantly decreased (*P* < 0.05). The above changes could be reversed by SYYQ intervention, and there was no significant difference in the results of Bcl-2 between groups (Figures 5G–I). These results suggest that CMD and Hyp can induce apoptosis and reduce the production of angiogenic factors, and SYYQ can reverse the above phenotypes.

### 3.5 SYYQ reverses the downregulation of miR-302a-3p and the activation of hippo pathway in hyp MCMECs

**FIGURE 6:**
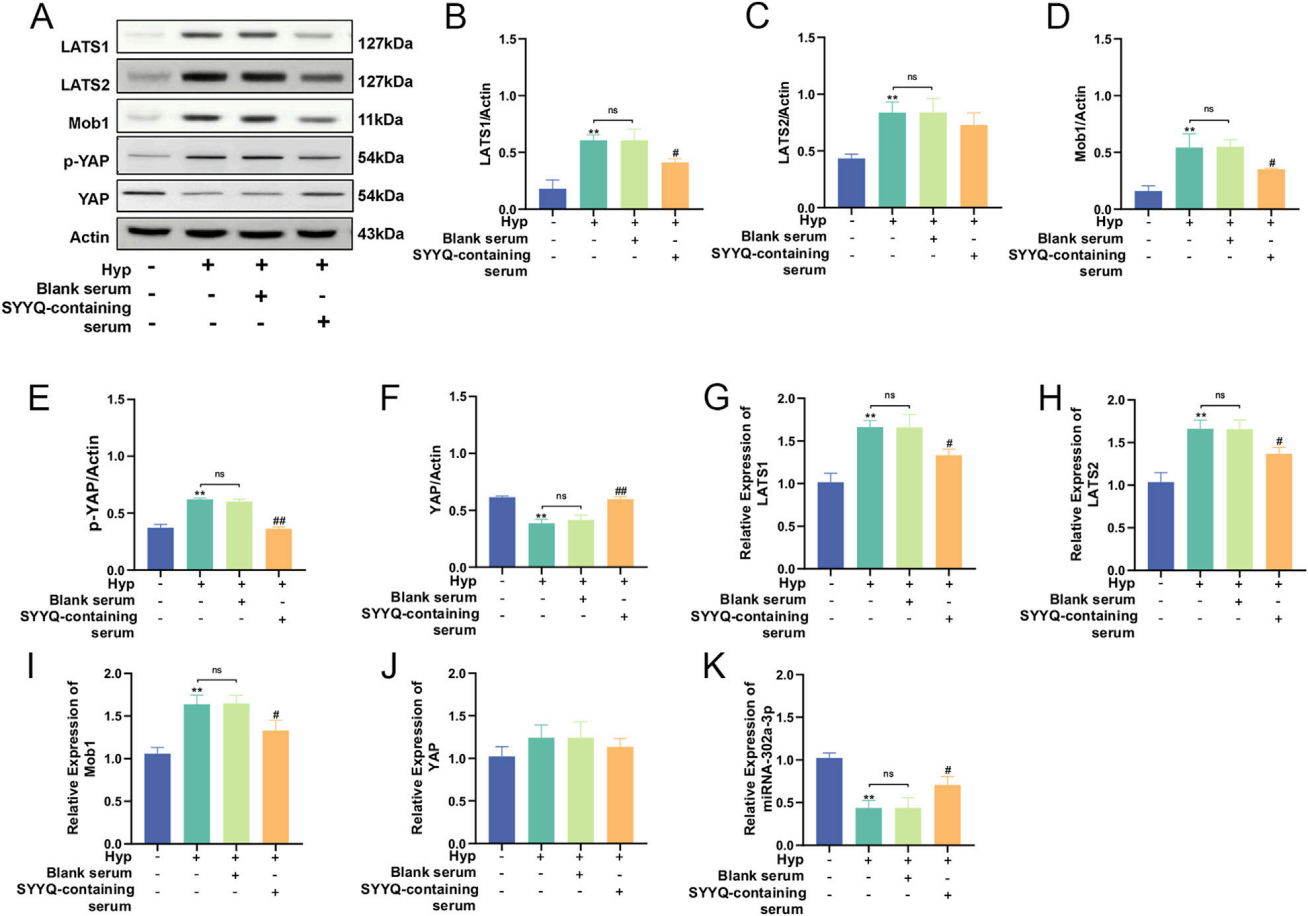
SYYQ reverses the downregulation of miR-302a-3p and the activation of Hippo pathway in Hyp MCMECs. Note: **(A–J)** Western blot and qRT-PCR were used to detect the expression of Hippo pathway related proteins. n = 3; **(K)** miR-302a-3p levels in each group *in vitro* n = 3. ^**/*^ *P* < 0.01/0.05 vs. Ctrl; ^#^ *P* < 0.05 vs. Hyp.

By utilizing high-throughput sequencing, Dai Rixin discovered that differential gene analysis and co-expression network analysis between patients with microvascular dysfunction in acute coronary syndrome and those with normal coronary artery flow primarily focused on the Hippo pathway, with miR-302a-3p being the most closely associated miRNA (Dai, 2022). In order to explore the changes of miR-302a-3p and Hippo pathways in Hyp MCMECs and the influence of SYYQ on them, WB and qRT-PCR were performed on MCMECs *in vitro*. The results showed that the level of miR-302a-3p in Hyp MCMECs decreased. The levels of LATS1/2, Mob1 and p-YAP were significantly increased, while the levels of YAP were significantly decreased (*P* < 0.05). SYYQ treatment reversed the above changes, and the differences in mRNA expression of YAP were not statistically significant (Figures 6A–K). These results indicated that Hyp decreased miR-302a-3p and activated Hippo signaling pathway in MCMECs, which could be reversed by SYYQ.

### 3.6 SYYQ may promote the generation of angiogenic factors and inhibit apoptosis in hyp MCMECs by inhibiting the hippo pathway

**FIGURE 7:**
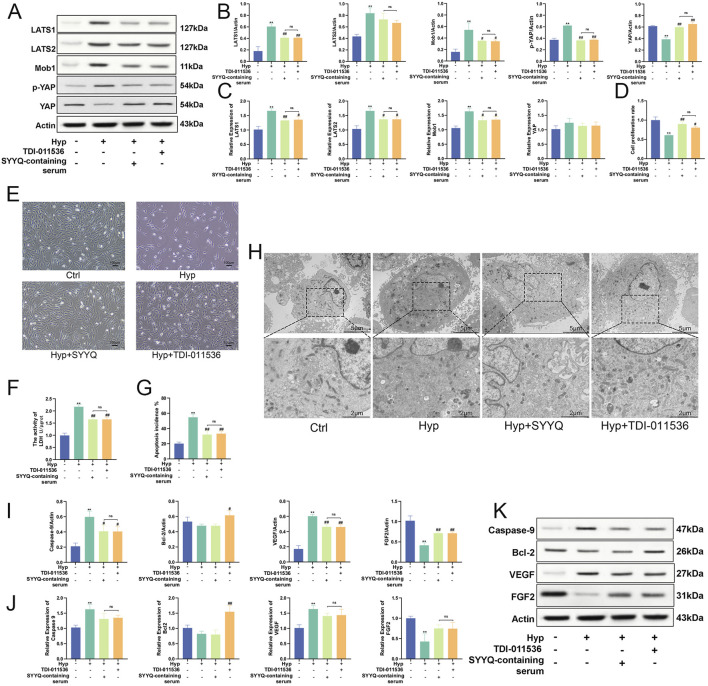
SYYQ may promote the generation of angiogenic factors and inhibit apoptosis in Hyp MCMECs by inhibiting the Hippo pathway. Note: **(A–C)** Hippo pathway related proteins expression was determined by Western blot and qRT-PCR. n = 3; **(D)** Cell viability assay; **(E)** Optical microscope images of MCMECs morphological changes; **(F)** Cell damage assay; **(G)** TUNEL assay to assess cell apoptosis rate *in vitro*. n = 3; **(H)** Transmission electron microscope images of MCMECs; **(I,K)** Caspase-9, Bcl-2, VEGF, FGF2 protein expression was determined by Western blot. n = 3; **(J)** Caspase-9, Bcl-2, VEGF, FGF2 mRNA level was assessed by qRT-PCR. n = 3. ^**^ *P* < 0.01 vs. Ctrl; ^##/#^ *P* < 0.01/0.05 vs. Hyp.

In light of the previously identified regulatory effects of SYYQ on the Hippo pathway, we aimed to investigate whether this effect is associated with its regulation of apoptosis and the production of angiogenic factors. For this purpose, we employed TDI-011536 to inhibit the Hippo pathway (Figures 7A–C). Our study showed that inhibition of Hippo pathway significantly increased the activity of Hyp MCMECs (*P* < 0.05) (Figures 7D,E) and reduced cell damage (*P* < 0.05) (Figure 7F). TEM results showed that inhibition of Hippo pathway had a similar effect on MCMECs as the intervention of SYYQ, which could alleviate Hyp-induced chromatin condensation, decrease nuclear membrane continuity, mitochondrial damage, and cytoplasmic vacuolar degeneration (Figure 7H). TUNEL staining indicated that the inhibition of the Hippo signaling pathway led to a decrease in apoptosis (*P* < 0.05) (Figure 7G; Supplementary Figure F3). WB results showed that inhibition of Hippo pathway significantly decreased the levels of Caspase-9 and VEGF protein in Hyp MCMECs (*P* < 0.05), and increased the levels of Bcl-2 and FGF2 (*P* < 0.05). The same trend was seen in qRT-PCR results. Similar to the effect of SYYQ on Hyp MCMECs (Figures 7I–K). These results suggest that SYYQ may promote the generation of angiogenic factors and inhibit apoptosis in Hyp MCMECs by inhibiting the Hippo pathway.

### 3.7 Overexpression of miR-302a-3p promotes the production of angiogenic factors and inhibits apoptosis induced by SYYQ *in vitro*

**FIGURE 8:**
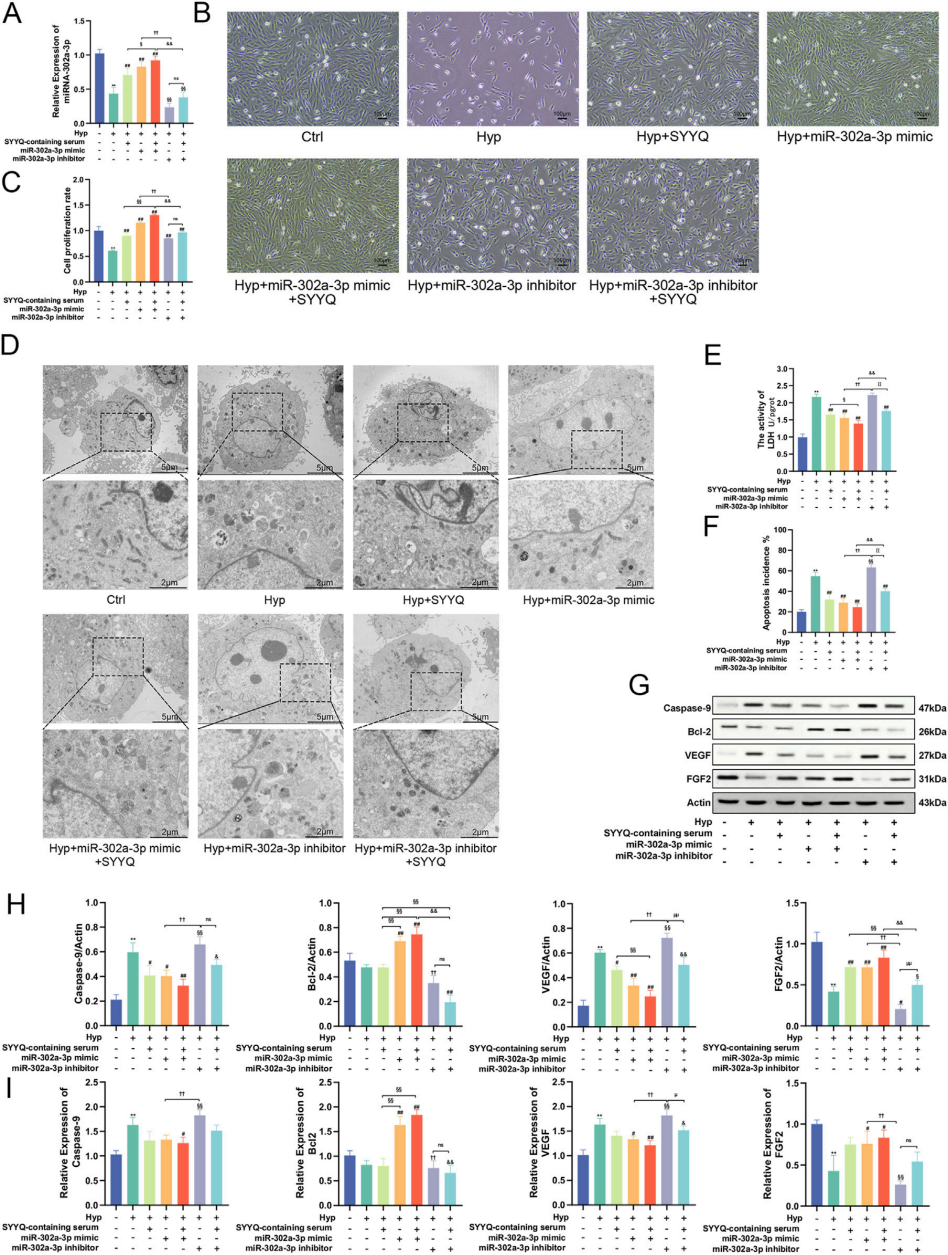
Overexpression of miR-302a-3p promotes the production of angiogenic factors and inhibits apoptosis induced by SYYQ *in vitro*. Note: **(A)** Levels of miR-302a-3p in each group *in vitro*. n = 3; **(B)** Optical microscope images of MCMECs morphological changes; **(C)** Cell viability assay; **(D)** Transmission electron microscope images of MCMECs; **(E)** Cell damage assay; **(F)** TUNEL assay to assess cell apoptosis rate *in vitro*. n = 3; **(G,H)** Caspase-9, Bcl-2, VEGF, FGF2 protein expression was determined by Western blot. n = 3; **(I)** Caspase-9, Bcl-2, VEGF, FGF2 mRNA level was assessed by qRT-PCR. n = 3. ^**^ *P* < 0.01 vs. Ctrl; ^##/#^ *P* < 0.01/0.05 vs. Hyp; ^§§^ *P* < 0.01 vs. Hyp + SYYQ; ^††^ *P* < 0.01 vs. Hyp + miR-302a-3p mimic; ^&&/&^ *P* < 0.01/0.05 vs. Hyp + miR-302a-3p mimic + SYYQ; ^μμ/μ^ *P* < 0.01/0.05 vs. Hyp + miR-302a-3p inhibitor.

Previous studies have shown the regulatory effect of SYYQ on miR-302a-3p, and we aimed to determine whether the regulatory effect of SYYQ is also related to the regulation of apoptosis and the production of angiogenic factors. The miR-302a-3p level was significantly increased after miR-302a-3p mimic transfection, and the miR-302a-3p level was significantly inhibited after miR-302a-3p inhibitor transfection of MCMECs cells (Figure 8A). Compared with the Hyp group, overexpression of miR-302a-3p significantly increased cell viability and reduced cell damage (*P* < 0.05). The intervention of SYYQ could enhance the above effects, and the silencing of miR-302a-3p had the opposite effect (Figures 8B,C,E). TEM results showed that overexpression of miR-302a-3p and SYYQ intervention both alleviated Hyp-induced chromatin condensation, mitochondrial swelling and blurriness, and cytoplasmic blister (Figure 8D). TUNEL staining showed that SYYQ administration and overexpression of miR-302a-3p resulted in decreased apoptosis, while silencing of miR-302a-3p increased apoptosis (*P* < 0.05) (Figure 8F; Supplementary Figure F4). WB results showed that overexpression of miR-302a-3p significantly decreased the protein levels of Caspase-9 and VEGF of Hyp MCMECs (*P* < 0.05), and increased the levels of Bcl-2 and FGF (*P* < 0.05). The intervention of SYYQ enhanced the above effects. Silencing miR-302a-3p had an opposite effect on the above proteins, and we saw the same trend in the qRT-PCR results (Figures 8G–I). The above results showed that overexpression of miR-302a-3p promoted the production of angiogenic factor and inhibited apoptosis induced by SYYQ *in vitro*.

### 3.8 SYYQ regulates the generation of angiogenic factors and inhibites apoptosis *in vitro* via miR-302a-3p/hippo

**FIGURE 9:**
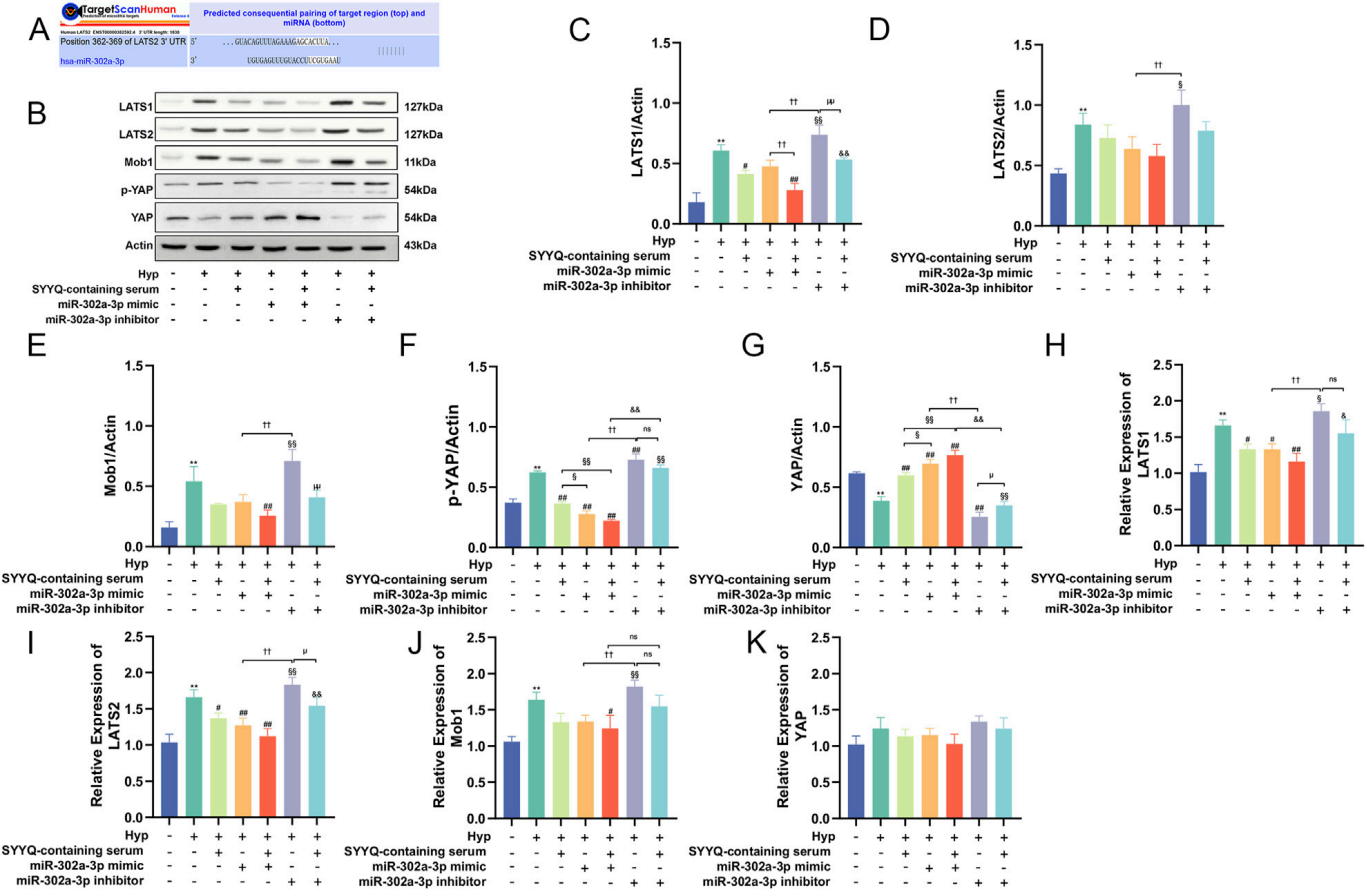
SYYQ regulates the generation of angiogenic factors and inhibits apoptosis *in vitro* via miR-302a-3p/Hippo. Note: **(A)** miR-302a-3p and LATS2 binding sites predicted by TargetScanHuman 8.0; **(B–K)** Hippo pathway related proteins expression was determined by Western blot and qRT-PCR. n = 3. ^**^ *P* < 0.01 vs. Ctrl; ^##/#^ *P* < 0.01/0.05 vs. Hyp; ^§§/§^ *P* < 0.01/0.05 vs. Hyp + SYYQ; ^††/†^ *P* < 0.01/0.05 vs. Hyp + miR-302a-3p mimic; ^&&/&^ *P* < 0.01/0.05 vs. Hyp + miR-302a-3p mimic + SYYQ; ^μμ/μ^ *P* < 0.01/0.05 vs. Hyp + miR-302a-3p inhibitor.

To predict potential downstream targets of miR-302a-3p, we found that LATS2 3‘UTR has a binding site for miR-302a-3p (Figure 9A). WB results showed that overexpression of miR-302a-3p decreased the levels of LATS1/2, Mob1, P-YAP, and increased the levels of YAP (*P* < 0.05). SYYQ intervention could enhance the above effects, whereas silencing miR-302a-3p reversed them (Figures 9B–K). These findings suggest that SYYQ promotes angiogenesis and inhibits apoptosis through the Hippo signaling pathway, mediated by miR-302a-3p.

## 4 Discussion

**FIGURE 10:**
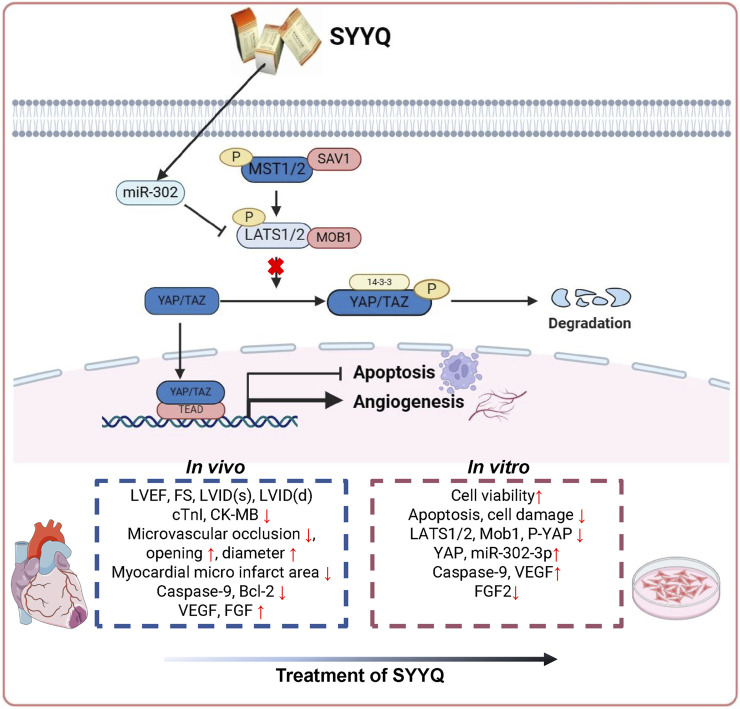
Schematic representation of proposed mechanism of SYYQ on the mitigative effect of CMD.

Based on the concept of multi-component and multi-target research, this study utilized network pharmacology to assess the potential mechanism of SYYQ intervention in CMD and validated it through *in vivo* and *in vitro* experiments. Network pharmacological analyses indicate that components such as Hirudinoidine A and quercetin may enhance CMD by influencing pathways associated with angiogenesis and apoptosis. Results from animal experiments suggest that SYYQ may mitigate myocardial injury in CMD rats by alleviating apoptosis, enhancing the production of angiogenic factors, decreasing microembolism formation, and improving coronary microvessel structure, with the most pronounced effect observed at high doses of SYYQ. Cell experiments demonstrated that SYYQ alleviated apoptosis and stimulated the production of angiogenic factors in Hyp MCMECs. Downregulation of miR-302a-3p levels and enhancement of the Hippo pathway were observed in Hyp MCMECs, a phenomenon reversible by SYYQ. When miR-302a-3p was overexpressed or the Hippo pathway was inhibited, SYYQ’s role in promoting the production of angiogenic factors and inhibiting apoptosis in Hyp MCMECs was further enhanced. Subsequent studies revealed that miR-302a-3p negatively regulates LATS2, with the main mechanism illustrated in Figure 10.

Despite the discovery of several drugs for the treatment of CMD in recent decades, the efficacy of these treatments in reducing the incidence of cardiovascular events remains unclear (Zhang et al., 2017b). Consequently, there is an urgent need to explore new and effective treatment strategies. TCM has developed a substantial repertoire of prescriptions for the treatment of CMD through extensive clinical practice. Tongxinluo capsule could enhance CMD by improving the structure and function of CMECs, protecting microvessels, enhancing vascular endothelial barrier function, and ameliorating endothelial dysfunction (Li et al., 2010; Qi et al., 2015; Qi et al., 2020; Zhang et al., 2015). Qishen Yiqi dropping pill effectively alleviates microcirculatory disturbances following ischemia-reperfusion while maintaining the integrity of the endothelial connections within the microvascular basement membrane (Lin et al., 2013; Pan et al., 2021). Shexiang Tongxin dropping pill can promote ischemic myocardial angiogenesis by promoting M1 macrophage polarization to alleviate cardiac function in CMD rats (Cui et al., 2023).

SYYQ is a TCM compound with the effect of tonifying qi and removing blood stasis. Previous studies indicate that SYYQ alleviates myocardial ischemia/reperfusion injury by mitigating endoplasmic reticulum stress (Tong et al., 2022), reducing apoptosis (Liu et al., 2013), and inhibiting mitochondrial autophagy activity (Zhang et al., 2023a). Additionally, it has been shown to alleviate atherosclerosis by enhancing autophagy (Lai et al., 2020; Zhou Y. et al., 2019). Recently, our research team conducted a randomized, single-blind, placebo-controlled study to evaluate the effects of SYYQ intervention on perioperative coronary microcirculation during PCI, utilizing the index of microcirculatory resistance (IMR) for evaluation (Li, 2020). The results demonstrated that SYYQ intervention significantly reduced the rate of IMR change before and after PCI, thereby providing clinical evidence supporting the efficacy of SYYQ in improving coronary microcirculation.

Currently, several methods are used to establish animal models of CMD, including: the metabolic disorder type, induced by drug interventions such as streptozotocin or alloxan, or through high-sugar and high-fat dietary regulation; the mechanical embolization type; the vascular endothelial injury type, caused by chemical agents. In mechanical embolization models, microspheres or autologous thrombi are commonly used. These microspheres are typically composed of chemically inert materials, such as polypropylene fibers, which lack biochemical activity. In contrast, clinical coronary microemboli consist of platelets, white blood cells, red blood cells, and atherosclerotic debris. While these components partially reflect clinical pathological changes, they do not represent *in situ* thrombi and fail to damage the microvascular endothelium, thus limiting their ability to accurately simulate the underlying causes of CMD. The metabolic disorder model often leads to systemic microvascular dysfunction, rather than being confined to the coronary microcirculation. Sodium laurate, however, exerts a strong endothelial-damaging effect. It can induce detachment and perforation of microvascular endothelial cells, increase coagulation factors, promote platelet adhesion and aggregation, and ultimately lead to microembolism formation. Therefore, in this study, sodium laurate was selected as the endothelial injury agent and administered via injection into the apex of the heart after aortic clamping, aiming to localize its effects primarily within the coronary circulation (Liu S. et al., 2021).

The dysfunction of CMECs is the key to the pathophysiology of CMD (Zhang et al., 2023b). CMECs regulate vasomotor function, maintain vascular permeability, and modulate angiogenesis in response to various stimulatory factors. Under specific pathological conditions, CMECs become damaged, leading to apoptosis, local inflammatory responses, and microvascular embolization, which collectively result in reduced coronary blood flow (Liu H. et al., 2021). A reduction in the number of myocardial capillaries is an important determinant of coronary blood flow reserve, and angiogenesis can effectively ameliorate CMD by enhancing blood supply capacity, restoring cardiac function, and improving hemodynamics (Yang et al., 2023). Consequently, identifying targets that regulate the apoptosis and angiogenesis of CMECs is of significant importance for treating CMD.

The Hippo signaling pathway is an important regulatory mechanism that governs the cell cycle and promotes angiogenesis (Azad et al., 2019; Rausch and Hansen, 2020). At the same time, its function in cardiovascular diseases has also been thoroughly studied. For example, Ma et al. (2020) found that inhibiting Hippo/YAP pathway by targeting LATS2 with miR-93 after myocardial infarction promoted angiogenesis and weakened myocardial remodeling. Liu S. et al. (2021) utilized gene therapy to knock down the Hippo pathway gene Salvador (Sav) in an ischemia/reperfusion pig model to promote myocardial cell renewal and enhance cardiac function. Wang et al. (2016) discovered that endothelial YAP/TAZ activity was influenced by varying blood flow patterns and was linked to endothelial dysfunction and the development of atherosclerosis. Consequently, the Hippo signaling pathway may represent a potential therapeutic target for CMD.

Apoptosis, also known as programmed cell death, is critical for tissue development and the maintenance of homeostasis. The apoptosis pathway involves two protein families: the Bcl-2 protein family and caspases. The former is considered the “extrinsic” pathway (related to Fas or TNF death receptors), and the latter is the “intrinsic” pathway (related to mitochondria) (Elmore, 2007). In this study, the biochemical results of serum Caspase-9 levels in animal experiments were entirely contrary to those of Bcl-2, whereas there was no significant difference in the WB and qRT-PCR results of Bcl-2 in cell experiments. TUNEL experiments indicated that SYYQ could inhibit apoptosis in CMD rats and Hyp MCMECs cells. The level of Bcl-2 increased following the inhibition of the Hippo pathway and the overexpression of miR-302a-3p, suggesting that SYYQ may not inhibit apoptosis in CMD rats and MCMECs via Bcl-2 pathway.

Angiogenesis is initiated by CMECs, which forms vascular networks through sprouting, branching, migration, and proliferation. Angiogenesis is regulated by a balance between angiogenic factors (including VEGF, angiopoietin, and FGF) and inhibitory factors (such as endostatin and angiostatin) (Zhou et al., 2024). VEGF controls cytoskeletal rearrangement, adhesion, and endothelial cells (ECs) polarization to facilitate the formation of new vessels (Chen et al., 2023). FGF promotes angiogenesis through ECs proliferation, migration, tube formation, and protease secretion (Javerzat et al., 2002). In this study, we found that SYYQ could induce VEGF and FGF in CMD rats and improve the patency of microvessels. However, this study did not specifically investigate whether collateral circulation was regenerated, a topic worthy of exploration in future research.

This study has several limitations: Firstly, we established an acute CMD rat model by clamping the aorta and injecting sodium laurate into the apex of the heart, which does not fully replicate the chronic nature of clinical lesion formation and does not mimic clinical comorbidities (e.g., diabetes, hypertension). Secondly, additional detection indicators are needed at the apoptosis level to better elucidate the effect of SYYQ on Hyp MCMECs apoptosis. Meanwhile, we did not investigate the impact of crosstalk between pathways on apoptosis. Thirdly, due to experimental limitations, all prototype components of SYYQ entering the bloodstream were not obtained via the ultra-high performance liquid chromatography-quadrupole time-of-flight mass spectrometry (UPLC/Q-TOF-MS) method, and YAP/TAZ’s role in angiogenesis/apoptosis is underexplored, and no high-throughput sequencing was conducted to confirm whether the key differential gene between CMD and patients with normal coronary microcirculation was miR-302a-3p, and this study did not establish another control group with a standard clinical treatment drug (e.g., statins or ACE inhibitors) of CMD. In addition, a dual-luciferase reporter assay was lacking to further validate the targeting relationship between miR-302a-3p and LATS2. Furthermore, SYYQ contains a variety of active ingredients, and its key compounds—such as Hirudinoidine A and quercetin—require further investigation into their effects on CMD.

## 5 Conclusion

In conclusion, SYYQ may improve CMD by promoting angiogenesis and inhibiting apoptosis, and its mechanism might involve the Hippo signaling pathway mediated by miR-302a-3p.

## Data Availability

The original contributions presented in the study are included in the article/Supplementary Material, further inquiries can be directed to the corresponding authors.
