# Supplementary material for: Mechanism of Shenyuan Yiqi Huoxue capsule alleviating coronary microvascular dysfunction: network analysis and experimental evidence
Source: Front Pharmacol. 2025 Aug 4;16:1534967. doi: 10.3389/fphar.2025.1534967 (PMC12358463; doi:10.3389/fphar.2025.1534967)
Supplement: Supplementary file 3 [file Supplementaryfile1.pdf]

Supplementary materials

Supplementary table S7 Initial processing of the Shenyuan Yiqi Huoxue Capsules (SYYQ)

Composition of SYYQ	Description of the extract and extraction process	Source
Huangqi**168g**, Dangshen**168g**, Xuanshen**84g**, Danshen**168g**, Shuizhi**112g**, Tubiecong**112g**, Dilong**56g**, Yanhusuo**168g**	Shuizhi and Dilong were ground into a fine powder. Huangqi, Dangshen, Xuanshen, and Tubiechong were decocted with water (mass: volume=1:10) three times, with each decoction lasting for 1 hour. The resulting decoctions were filtered, combined, and concentrated to a final volume ratio of 1:1. Two volumes of ethanol were added to the concentrated solution, followed by thorough mixing and standing at 5 °C for 16 hours. The mixture was then filtered again, and the supernatant was reserved for further processing. Danshen and Yanhusuo were extracted three times using 10 volumes of 75% ethanol under reflux heating, with each extraction lasting 1.5 hours. The extracts were filtered to obtain an alcohol precipitation filtrate. This filtrate was combined with the previously obtained aqueous filtrate, and the ethanol was removed under reduced pressure. The mixture was concentrated to a relative density of 1.25 (measured at 60 °C), dried, and ground into a fine powder. The powder was then mixed thoroughly with the Shuizhi and Dilong fine powder, pelleted using 85% ethanol, encapsulated, and processed into 1000 capsules	All medicinal herbs were purchased from Beijing Xinglin Pharmaceutical Co. (Beijing, China) and were authenticated by Kechen Mao, a professional herbalist from Beijing TCM Hospital, Capital Medical University.<br><br>We hereby confirm that the collection and processing of plant materials for this study fully comply with the Nagoya Protocol, CITES, all associated treaties including phytosanitary regulations, as well as the laws and regulations of China and the requirements of the Chinese Pharmacopoeia.

Supplementary material 1 Oral Acute Toxicity Test of SYYQ (Maximum Administered Dose)

（1）Experimental methods

Mice were fasted for 16 hours before administration and were not provided with water during this period. During the experiment, the animals were randomly assigned to either a negative control group or an SYYQ-treated group based on their body weight. The negative control group received sterile drinking water at a dose of 0.4 mL/10 g body weight, while the SYYQ group was administered the compound via gavage at a dose of 0.64g(0.4mL)/10g body weight. Administration was performed twice daily, in the morning and afternoon, resulting in a total daily dosage of 128.00g/kg for the SYYQ group, which corresponds to approximately 412.90 times the clinical dosage. The immediate reaction following administration was observed, as well as general behavior and body weight changes after 1 week and 2 weeks of treatment.

（2）Experimental result

Mice were administered SYYQ via gavage at the maximum allowable concentration and administration volume. No abnormal activity or adverse reactions were observed following administration. After 14 days of continuous observation, the mice exhibited smooth fur and skin, normal feeding behavior and activity levels, and steady weight gain. There was no statistically significant difference in body weight between the SYYQ group and the negative control group before or after treatment.

Table 1 The effect of SYYQ on mice body weight ($\bar{\chi}$±s,n=10)

Group	Sex	Before administration	One week after administration	Two weeks after administration
Negative Control	Female	19.05±0.57	23.60±1.04	26.22±1.13
	Male	19.22±0.49	28.95±2.18	31.92±1.86
SYYQ	Female	19.14±0.61	22.85±1.01	25.68±0.80
	Male	19.10±0.69	27.40±1.08	30.70±1.30

Table 2 Acute Toxicity Test Results of SYYQ ($\bar{\chi}$±s,n=20)

Group	Immediate reaction after administration

	Appearance	Limb movement	Ingestion	Drinking water	Excretion	Death
Negative Control	-	-	-	-	-	0
SYYQ	-	-	-	-	-	0

Note: “-” is normal

Table 3 Acute Toxicity Test Results of SYYQ ($\bar{\chi}$±s,n=20)

Group	Reaction during observation after administration					
	Appearance	Limb movement	Ingestion	Drinking water	Excretion	Death
Negative Control	-	-	-	-	-	0
SYYQ	-	-	-	-	-	0

Note: “-” is normal

（3）Conclusion

Mice were orally administered SYYQ at a dose of 128.00 g/kg, equivalent to approximately 412.90 times the clinical dosage. No toxic reactions were observed following 14 days of post-administration monitoring.

Supplementary material 2 The quality control and pharmacodynamic study of the SYYQ

The quality control of the SYYQ

We used ultra performance liquid chromatography quadrupole-time of flight mass spectrometry (UPLC/Q-TOF-MS/MS) to investigate the chemical composition and content determination of SYYQ. We accurately weighed 5.0mg SYYQ capsule powder and added it to 0.5mL of 80% methanol-20% aqueous solution. After stirring the above mixture evenly, sonicate for 15 minutes and centrifuge at 12000r/min for 15 minutes. Take the supernatant and filter it through a 0.45 μ m membrane to obtain the detection sample of SYYQ capsules.

Chromatography and mass spectrometry conditions:

Liquid phase conditions: The chromatographic column is SHIMADZU InerSustain C18 (100×2.1mm, 2 μ m); The column temperature is 35℃; The mobile phase consists of acetonitrile (A) and 0.1% formic acid aqueous solution (B); The flow rate is 1mL • min^{-1}; Gradient elution conditions: 5% A for 0-2 minutes; 2~4min 5~20%A； 4~12min 20~25%A； 12~14min 25~46%A； 14~26min 46~0%A； 26~28min 100%A； 28~30min 100~5%A； The injection volume is 10 μ L.

Mass spectrometry conditions: The positive ion and negative ion modes of electric spray ionization (ESI) are used for detection. The ESI source conditions are as follows: ion source gas 1 (Gas 1): 50, ion source gas 2 (Gas2): 50, curtain gas (CUG): 25, source temperature: 500℃ (positive ion) and 450℃ (negative ion), ion source voltage (ISVF) 5500V (positive ion) and 4400V (negative ion), TOF MS scanning range: 100-1200Da, product example scanning range: 50-1000Da, TOF MS scanning cumulative time 0.2s, product example scanning cumulative time 0.01s, and secondary mass spectrometry uses data dependence Type IDA scanning, and high sensitivity mode is adopted, clustering voltage (DP): ±60V, collision energy: 35±15eV.

The results of UPLC chromatographic analysis：

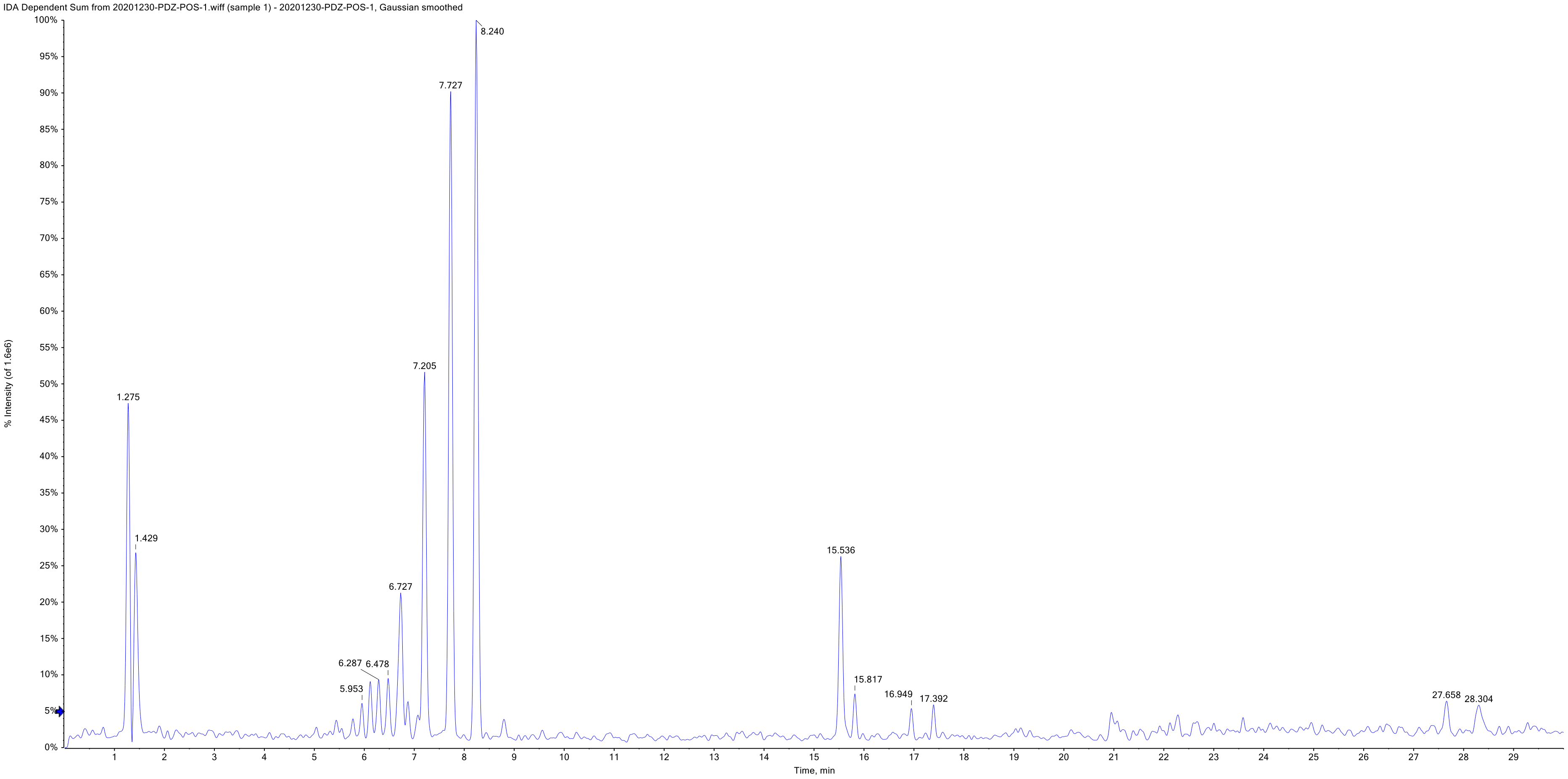

Fig. 1 SYYQ positive ion mode chromatogram

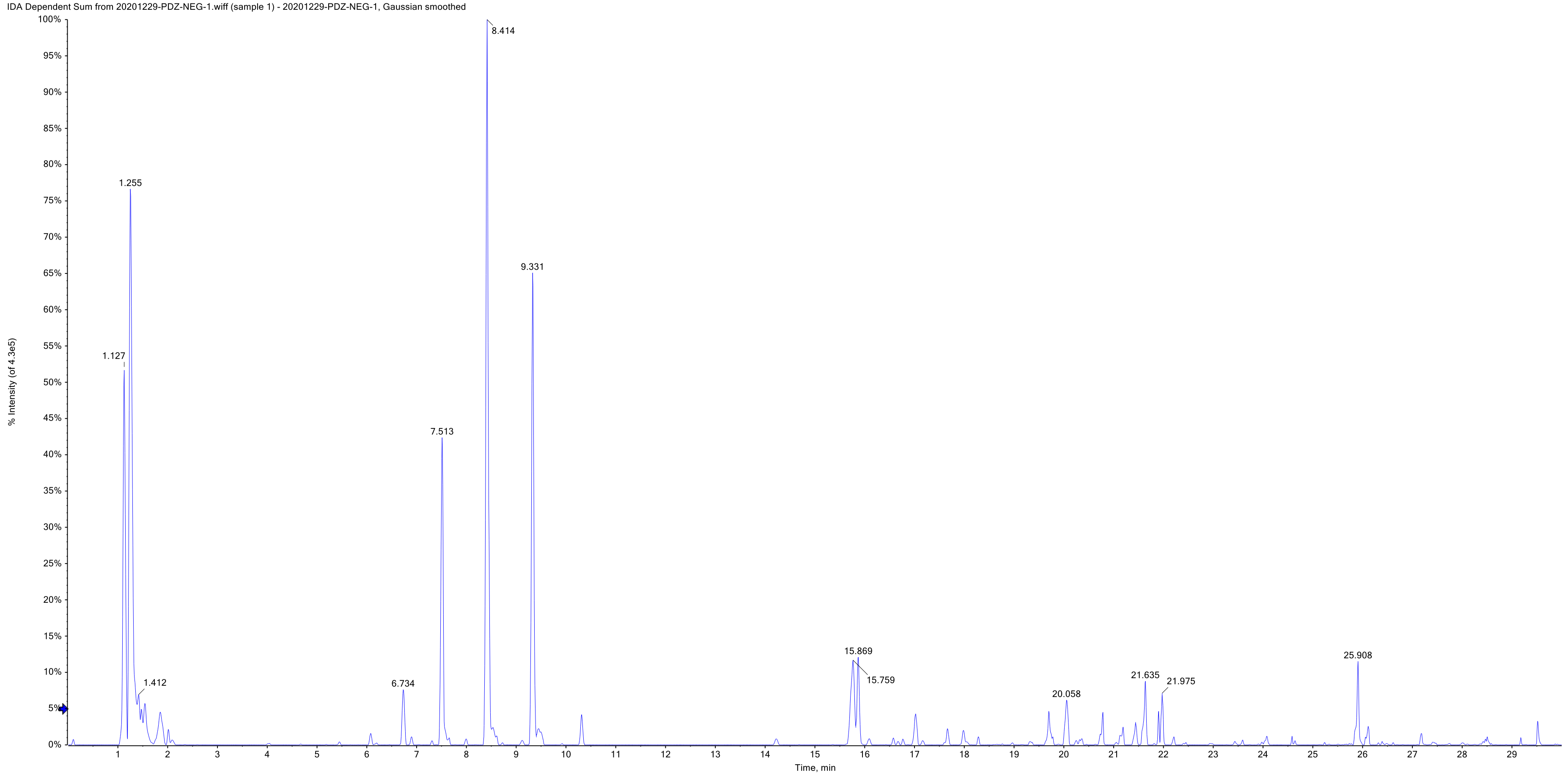

Fig. 2 SYYQ negative ion mode chromatogram

Each capsule of this product contains not less than 0.0500mg of Huangqi (*Astragalus mongholicus* Bunge) calculated as Isoflavones Glucoside (C22H22O10), and not less than 0.130mg of Danshen (*Salvia miltiorrhiza* Bunge) calculated as Tanshinone IIA (C19H18O3). Meanwhile, We

found that the components obtained from the network pharmacology analysis, such as Tanshinone IIA, formononetin, Calycosin, 4-Hydroxy-3,5-dimethoxycinnamic acid, berberine, dehydrocavidine, Phenylalanine, leonticine, Stearic Acid, astragaloside iv, tetramethyl monoxide, Calycosin, (R)-Canadine, and coptisine, are indeed present in SYYQ.

The pharmacodynamic study of the SYYQ

Methods:

24 healthy rats were randomly divided into SYYQ group, Astragalus monotherapy group, Salviamiltiorrhiza monotherapy group and Yanhuso monotherapy group. They were fasted withoutwater 12h before the experiment and given acertainamount of liguid medicineintragastrically at 5min, 15min, 0.5h, lh, 1.5h, 2h, 3h, 4h, 6h, 8h, 12h, 16h and 24h to getblood from the eyes.Plasma was treated by isopropanol protein precipitation method, and theblood drugconcentration ofeachsample ateachtime pointwas determined byUPLC-MS/MS technology. Phoenix Winnonlin8.1 software was used to calculate the mainpharmacokinetic parameters and draw the drug time curve. SPSS20.0 software was used forstatistical analysis.

Results:

Table 1 Pharmacokinetic parameters of different groups of the three analytes

	Group	T_{max}（h）	C_{max}（ng/mL）	AUC_{0-t}(ng/mL*h)	$AUC_{0-\infty}$(ng/mL*h)	$T_{1/2}$(h)	MRT_{0-t} (h)
astragaloside iv	SYYQ (n=6)	4.50±2.93*↑	1.19±0.78**↑	86.14±48.15*↑	28.61±15.42**↑	9.21±7.51*↑	5.15±1.94**↑
	Astragalus monotherapy group	0.82±0.74	1.03±0.31	11.45±5.19	7.88±4.45	2.16±0.81	2.36±0.75

	(n=5)						
Tanshinone IIA	SYYQ (n=6)	2.58±2.99	3.29±1.16	313.6±423.17*↓	98.3±39.62*↓	2.58±2.99**↑	7.03±5.93
	Salviamiltiorrhiza monotherapy group (n=5)	0.60±0.38	12.34±11.04	475.95±582.18	325.78±343.68	0.60±0.384	4.52±2.15
tetramethyl monoxide	SYYQ (n=6)	0.71±0.33	51.64±17.14**↓	1197.71 ± 195.90**↓	835.12±213.40**↓	5.20±1.05**↓	4.96±1.42**↓
	Yanhuso monotherapy group (n=6)	0.38±0.14	59.57±19.25	3808.67±3271.37	1174.04±676.96	14.06±7.19	6.71±1.70

Note: Compared with the single drug group administered by gavage: ***P* < 0.01； **P* < 0.05

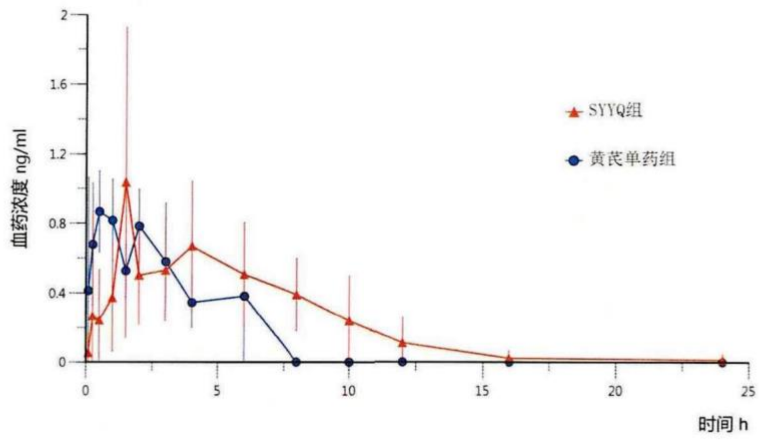

Fig. 3 Time curve of Astragaloside IV during oral administration of SYYQ and Astragalus monotherapy

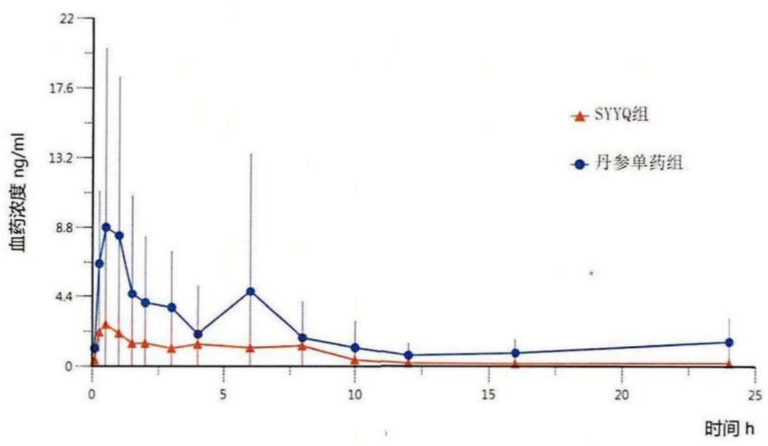

Fig. 4 Time curve of Tanshinone IIA during oral administration of SYYQ and Salviamiltiorrhiza monotherapy

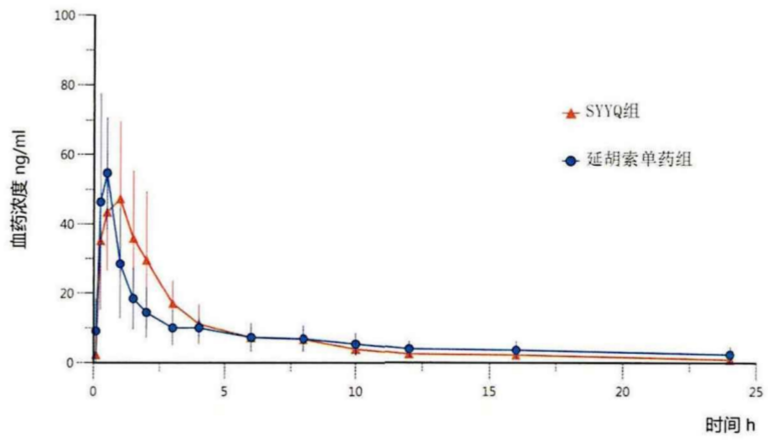

Fig. 5 Time curve of tetrahydropalmatine during oral administration of SYYQ and Yanhusuo monotherapy

Compared withthe single drug group, the T_{max} of astragaloside IV in SYYQ compound was delayed compared with the single drug group. There was no statistical difference between the T_{max} results of tanshinone II A and tetramyoxin. The absorption and distribution of astragaloside IV and tanshinone PSA showed A double peak phenomenon, and the C_{max} and AUC of astragaloside IV in SYYQ group were higher than those in the single drug group. The AUC and C_{max} of tanshinone II A and tetramethyl monoxide in SYYQ group were lower than those in monotherapy group. The MRT_{0-t} and $T_{1/2}$ of astragaloside lV and $T_{1/2}$ of tanshinone PSA in SYYQ group were longer than those in monotherapy group, and the MRT_{0-t} and $T_{1/2}$ of tetramethyl monoxide were shorter than those in monotherapy group.

Conclusions:

The pharacokinetic behavior of some components of SYYQ were preliminary investigated, and it was found that the absorption and exposure degree of some components were higher in the compound, and the compatibility of traditional Chinese medicines could increase or shorten the action time of some components, so as to achieve the purpose of slow release or reduce the toxic and side effects.

Supplementary material 3 The culturing principle of the mice coronary microvascular endothelial cells (MCMECs)

To simulate the pathophysiological mechanisms of coronary microvascular disorder (CMD), this study employed a three-gas hypoxic culture system (1%Air-94%N_2-5%CO_2) to induce a 12-hour hypoxic intervention in MCMECs. This model replicates the pathological features of clinical coronary microcirculatory dysfunction by precisely controlling the oxygen environment. The mechanism of action is as follows: under hypoxic conditions, prolyl hydroxylase domain (PHD) enzyme activity is inhibited, leading to the stabilization and accumulation of hypoxia-inducible factor-1α (HIF-1α) protein[1]. HIF-1α subsequently activates angiogenesis-related signaling pathways such as VEGF and ANG-2[2], while also inducing mitochondrial dysfunction[3] and promoting the release of inflammatory cytokines[4]. Collectively, these effects result in impaired endothelial barrier integrity, dysregulated vasomotor function, and a prothrombotic tendency. This model successfully recapitulates the key features of CMD, including microvascular endothelial dysfunction, perfusion abnormalities, and the pathological processes associated with myocardial ischemia.

[1]Wang J, Zhou J, Wang Y, Yang C, Fu M, Zhang J, Han X, Li Z, Hu K, Ge J. Qiliqiangxin protects against anoxic injury in cardiac microvascular endothelial cells via NRG-1/ErbB-PI3K/Akt/mTOR pathway. J Cell Mol Med, 2017,21(9):1905-1914.

[2]Wang Y, Zhang J, Fu M, Wang J, Cui X, Song Y, Han X, Liu Y, Zhou J, Ge J. Qiliqiangxin Prescription Promotes Angiogenesis of Hypoxic Primary Rat Cardiac Microvascular Endothelial Cells via Regulating miR-21 Signaling. Curr Pharm Des, 2021,27(26):2966-2974.

[3]Xiao X, Xu S, Li L, Mao M, Wang J, Li Y, Wang Z, Ye F, Huang L. The Effect of Velvet Antler Proteins on Cardiac Microvascular Endothelial Cells Challenged with Ischemia-Hypoxia. Front Pharmacol, 2017,8:601.

[4]Li YN, Wang XJ, Li B, Liu K, Qi JS, Liu BH, Tian Y. Tongxinluo inhibits cyclooxygenase-2, inducible nitric oxide synthase, hypoxia-inducible factor-2α/vascular endothelial growth factor to antagonize injury in hypoxia-stimulated cardiac microvascular endothelial cells. Chin Med J (Engl), 2015,128(8):1114-1120.

Supplementary table S8 Echocardiographic results of rats in each group($\bar{\chi}$±s,n=6)

Group	LVDd(mm)	LVDs(mm)	FS(%)	LVEF(%)
Sham	8.2±0.3	4.2±0.5	45.78±7.0	75.0±7.8
CMD	6.0±0.5	3.9±0.8	35.97±1.9	64.7±2.6
SYYQ-L	6.3±0.2	3.5±0.8	46.53±9.4	76.1±10.2
SYYQ-M	5.8±0.4	3.2±0.5	45.09±6.4	76.7±5.8
SYYQ-H	6.0±0.4	3.0±0.7	49.72±8.2	79.8±8.3
Nicorandil	6.2±0.3	4.1±1.1	36.14±3.6	64.7±5.2

Supplementary table S9 CK-MB results of rats in each group at baseline, 4h, 24h and 7d after operation($\bar{\chi}$±s,n=6)(ng/mL)

Group	baseline	4h	24h	7d
Sham	30.1±1.2	30.2±0.5	31.9±1.4	31.7±0.6
CMD	31.7±0.8	52.0±0.9**	68.2±1.4**	72.2±1.3**
SYYQ-L	29.9±1.3	36.3±1.3$^{\#\#}$	30.5±0.9$^{\#\#\S\S}$	27.7±1.0$^{\#\#\S\S}$
SYYQ-M	20.9±1.5	39.4±1.0	31.1±0.6$^{\#\#\S\S}$	28.4±1.2$^{\#\#\S\S}$
SYYQ-H	30.8±1.1	37.1±1.0$^{\#}$	24.6±1.0$^{\#\#\S\S\dagger\dagger}$	20.0±1.0$^{\#\#\S\S\dagger\dagger}$
Nicorandil	31.2±0.6	37.6±0.7	27.8±1.4$^{\#\#}$	25.5±0.9$^{\#\#}$

Note：$^{**}P$＜0.01, $^{*}P$＜0.05 *vs* Sham group; $^{\#\#}P$＜0.01, $^{\#}P$＜0.05 *vs* CMD group; $^{\S\S}P$＜0.01, $^{\S}P$＜0.05 *vs* Nicorandil group; $^{\dagger\dagger}P$＜0.01 *vs* SYYQ-L group

Supplementary table S10 cTnI results of rats in each group at baseline, 4h, 24h and 7d after operation($\bar{\chi}$±s,n=6)(ng/L)

Group	baseline	4h	24h	7d
Sham	69.6±1.4	71.2±1.2	71.7±1.1	70.3±0.9
CMD	71.7±0.9	87.9±1.4**	116.9±1.3**	130.1±1.5**
SYYQ-L	70.8±1.3	72.7±1.4$^{\#\#}$	61.2±0.7$^{\#\#}$	57.2±1.2$^{\#\#\S}$
SYYQ-M	70.3±1.9	72.4±1.3$^{\#\#}$	58.0±1.1$^{\#\#\dagger\dagger}$	56.5±1.6$^{\#\#}$
SYYQ-H	69.8±2.3	72.9±0.7$^{\#\#}$	48.7±1.3$^{\#\#\S\S\dagger\dagger}$	39.0±1.8$^{\#\#\S\S\dagger\dagger}$
Nicorandil	71.4±1.5	74.3±2.3$^{\#\#}$	59.5±1.8$^{\#\#}$	54.2±2.0$^{\#\#}$

Note：$^{**}P$＜0.01 *vs* Sham group; $^{\#\#}P$＜0.01 *vs* CMD group; $^{\S\S}P$＜0.01, $^{\S}P$＜0.05 *vs* Nicorandil group; $^{\dagger\dagger}P$＜0.01 *vs* SYYQ-L group

Supplementary table S11 Apex microvascular occlusion rate of rats in each group($\bar{\chi}$±s,n=3)

Group	microvascular occlusion rate(%)
Sham	0
CMD	76.0±13.1*

SYYQ-L	33.0±19.2#
SYYQ-M	27.5±16.0#
SYYQ-H	15.5±15.2#
Nicorandil	32.3±23.7#

Note：*$P<0.05$ *vs* Sham group, #$P<0.05$ *vs* CMD group, §$P<0.05$ *vs* Nicorandil group, †$P<0.05$ *vs* SYYQ-L group

Supplementary table S12 Apex microvascular opening rate of rats in each group($\bar{\chi}$±s,n=3)

Group	microvascular opening rate(%)
Sham	100.0
CMD	24.0±13.1*
SYYQ-L	67.0±19.2#
SYYQ-M	72.5±16.0#
SYYQ-H	84.6±15.2#
Nicorandil	67.7±23.7#

Note：*$P<0.05$ *vs* Sham group, #$P<0.05$ *vs* CMD group

Supplementary table S13 Coronary microvascular diameter of rats in each group($\bar{\chi}$±s,n=3)

Group	microvascular diameter(μm)
Sham	71.0±23.9
CMD	46.0±20.5*
SYYQ-L	60.3±18.4
SYYQ-M	64.9±19.2#
SYYQ-H	69.1±22.2#
Nicorandil	61.3±17.6#

Note：*$P<0.05$ *vs* Sham group, #$P<0.05$ *vs* CMD group

Supplementary table S14 The area ratio of Heidenhain positive expression in cardiac tissue($\bar{\chi}$±s,n=3)

Group	The are ratio of micro infarction(%)
Sham	0
CMD	12.4±2.2*
SYYQ-L	3.1±0.9
SYYQ-M	1.9±0.6#
SYYQ-H	1.8±0.3#
Nicorandil	5.1±1.6

Note：*$P<0.05$ *vs* Sham group, #$P<0.05$ *vs* CMD group

Supplementary table S15 Statistical analysis of the percentage of TUNEL-positive nuclei($\bar{\chi}$±s， n=6)

Group	TUNEL Positive Myocytes(%)
Sham	0.07±0.04
CMD	0.11±0.03*
SYYQ-L	0.10±0.01
SYYQ-M	0.10±0.01
SYYQ-H	0.09±0.01
Nicorandil	0.11±0.01

Note：$^{*}P$＜0.05 *vs* Sham group

Supplementary table S16 Caspase-9 results of rats in each group at baseline, 4h, 24h and 7d after operation($\bar{\chi}$±s,n=6)(ng/L)

Group	baseline	4h	24h	7d
Sham	2.2±0.2	2.6±0.4	2.2±0.4	2.3±0.2
CMD	2.5±0.4	3.4±0.4**	4.6±0.2**	5.0±0.3*
SYYQ-L	2.2±0.2	3.1±0.3	3.2±0.4	2.4±0.3
SYYQ-M	2.1±0.4	3.2±0.1	2.8±0.2	2.2±0.3$^{\#}$
SYYQ-H	2.1±0.1	2.8±0.2$^{\#\#}$	2.5±0.1$^{\#\#}$	1.7±0.3$^{\S\#\#}$
Nicorandil	2.2±0.1	3.1±0.2	2.7±0.2$^{\#}$	2.4±0.1

Note：$^{**}P$＜0.01, $^{*}P$＜0.05 *vs* Sham group; $^{\#\#}P$＜0.01, $^{\#}P$＜0.05 *vs* CMD group; $^{\S}P$＜0.05 *vs* Nicorandil group

Supplementary table S17 Bcl-2 results of rats in each group at baseline, 4h, 24h and 7d after operation($\bar{\chi}$±s,n=6)(ng/L)

Group	baseline	4h	24h	7d
Sham	9.0±1.1	9.7±0.7	9.9±0.7	9.8±0.3
CMD	9.47±0.4	13.9±0.6*	14.7±1.0*	15.1±0.3*
SYYQ-L	8.95±0.9	9.2±0.6$^{\#\#\S}$	8.5±0.7$^{\#\#}$	8.0±1.4$^{\#\#}$
SYYQ-M	9.50±0.4	9.4±0.2$^{\#}$	8.7±0.8$^{\#\#}$	7.8±0.6$^{\#\#}$
SYYQ-H	9.0±0.7	9.1±0.8$^{\#\#}$	8.2±0.3$^{\#\#\S\S}$	6.9±1.2$^{\#\#\S}$
Nicorandil	8.8±1.2	12.1±0.5	11.4±0.8	9.9±0.1

Note：$^{*}P$＜0.05 *vs* Sham group; $^{\#\#}P$＜0.01, $^{\#}P$＜0.05 *vs* CMD group; $^{\S\S}P$＜0.01, $^{\S}P$＜0.05 *vs* Nicorandil group

Supplementary table S18 VEGF results of rats in each group at baseline, 4h, 24h and 7d after operation($\bar{\chi}$±s,n=6)(ng/L)

Group	baseline	4h	24h	7d
Sham	70.3±1.3	70.1±1.0	71.7±1.5	70.4±1.1
CMD	70.8±0.2	80.7±1.5**	88.1±2.3**	96.4±2.9*
SYYQ-L	71.2±0.3	79.7±1.2	82.0±0.5$^{\#\#\S\S}$	81.9±0.8
SYYQ-M	71.0±0.3	82.2±0.7††	**88.0±1.8**§§††	103.2±3.0
SYYQ-H	71.1±0.3	83.0±1.9$^{\#\dagger\dagger}$	104.1±1.3$^{\#\#\S\S\dagger\dagger}$	116.8±1.5$^{\#\dagger\dagger}$

Nicorandil	71.0±0.2	81.8±0.9	91.3±1.2##	102.8±1.1

Note：**P<0.01, *P<0.05 *vs* Sham group; ##P<0.01, #P<0.05 *vs* CMD group; §§P<0.01 *vs* Nicorandil group; ††P<0.01 *vs* SYYQ-L group

Supplementary table S19 FGF results of rats in each group at baseline, 4h, 24h and 7d after operation($\bar{\chi}$±s,n=6)(ng/L)

Group	baseline	4h	24h	7d
Sham	212.6(205.6, 214.3)	213.1(207.9, 215.2)	210.5±4.7	211.2(208.7, 212.9)
CMD	209.3(203.9, 214.4)	230.8(218.6, 238.7)*	250.9±1.9**	261.6(258.5, 263.4)*
SYYQ-L	209.1(205.7, 211.9)	245.7(242.6, 248.8)	269.8±3.9##§†	273.7(265.2, 274.3)
SYYQ-M	217.6(210.2, 219.7)	242.1(238.3, 245.0)	**281.5±6.2**##	294.8(289.8, 308.7)
SYYQ-H	213.5(210.8, 215.4)	257.9(254.6, 262.9)#	312.8±4.6##§§††	324.5(306.7, 328.3)##†
Nicorandil	209.6(207.6, 236.1)	255.1(251.4, 265.0)	280.6±8.7##	299.6(297.3, 305.4)

Note：**P<0.01, *P<0.05 *vs* Sham group; ##P<0.01, #P<0.05 *vs* CMD group; §§P<0.01, §P<0.05 *vs* Nicorandil group; ††P<0.01, ††P<0.01, †P<0.05 *vs* SYYQ-L group

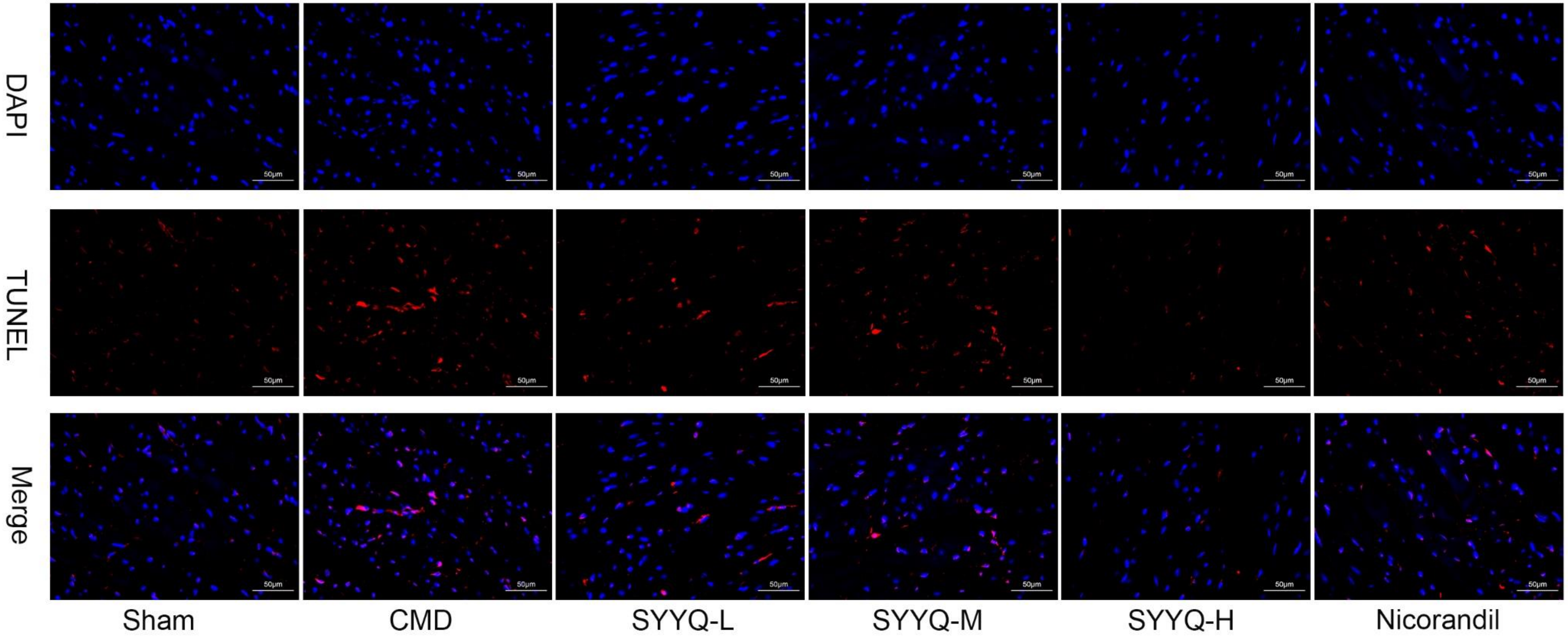

Supplementary figure F1 TUNEL assay was used to determine the apoptosis rate in *vivo* (n = 6)

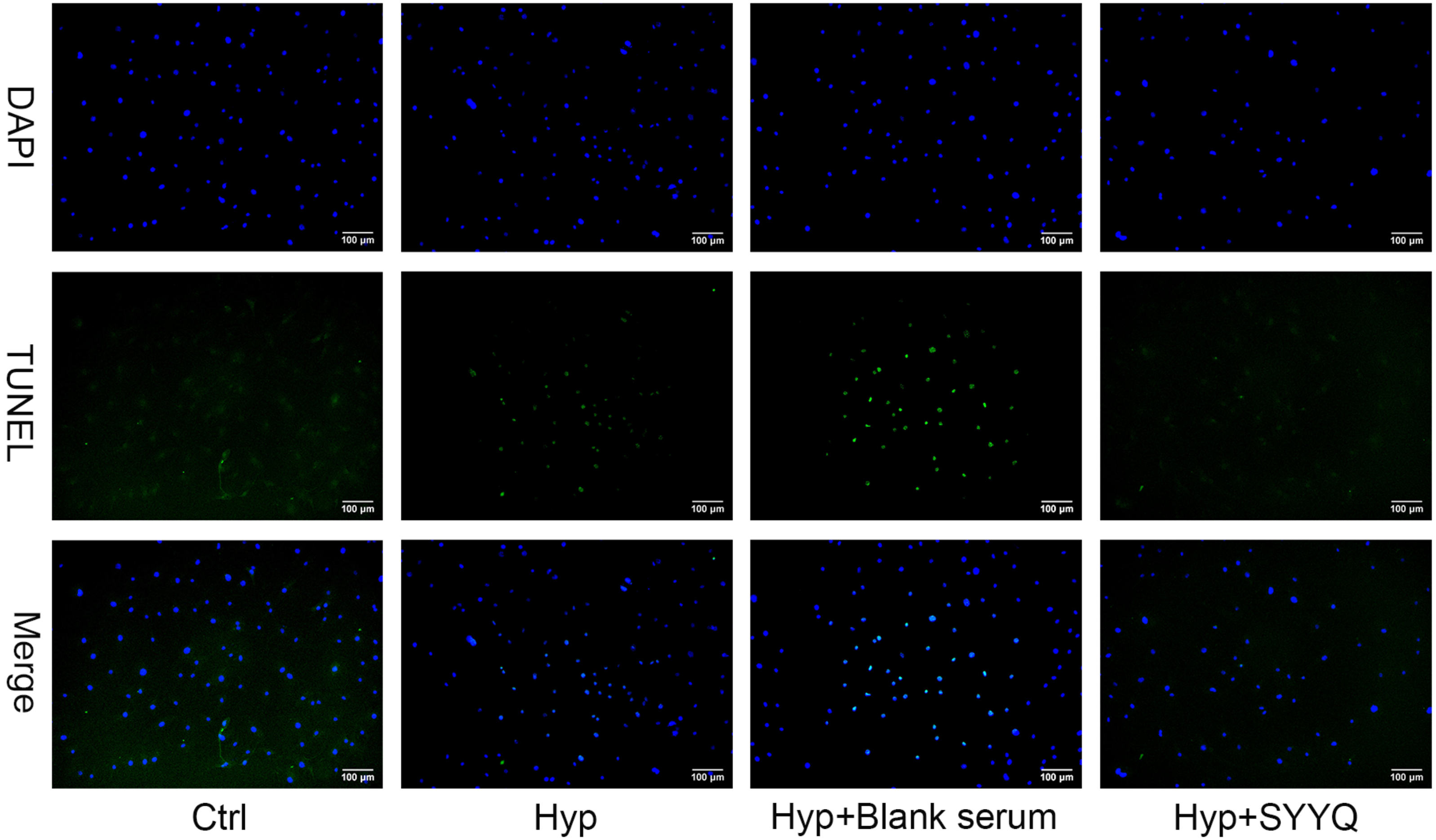

Supplementary figure F2 The apoptosis rate was measured *in vitro* by TUNEL assay (n=3)

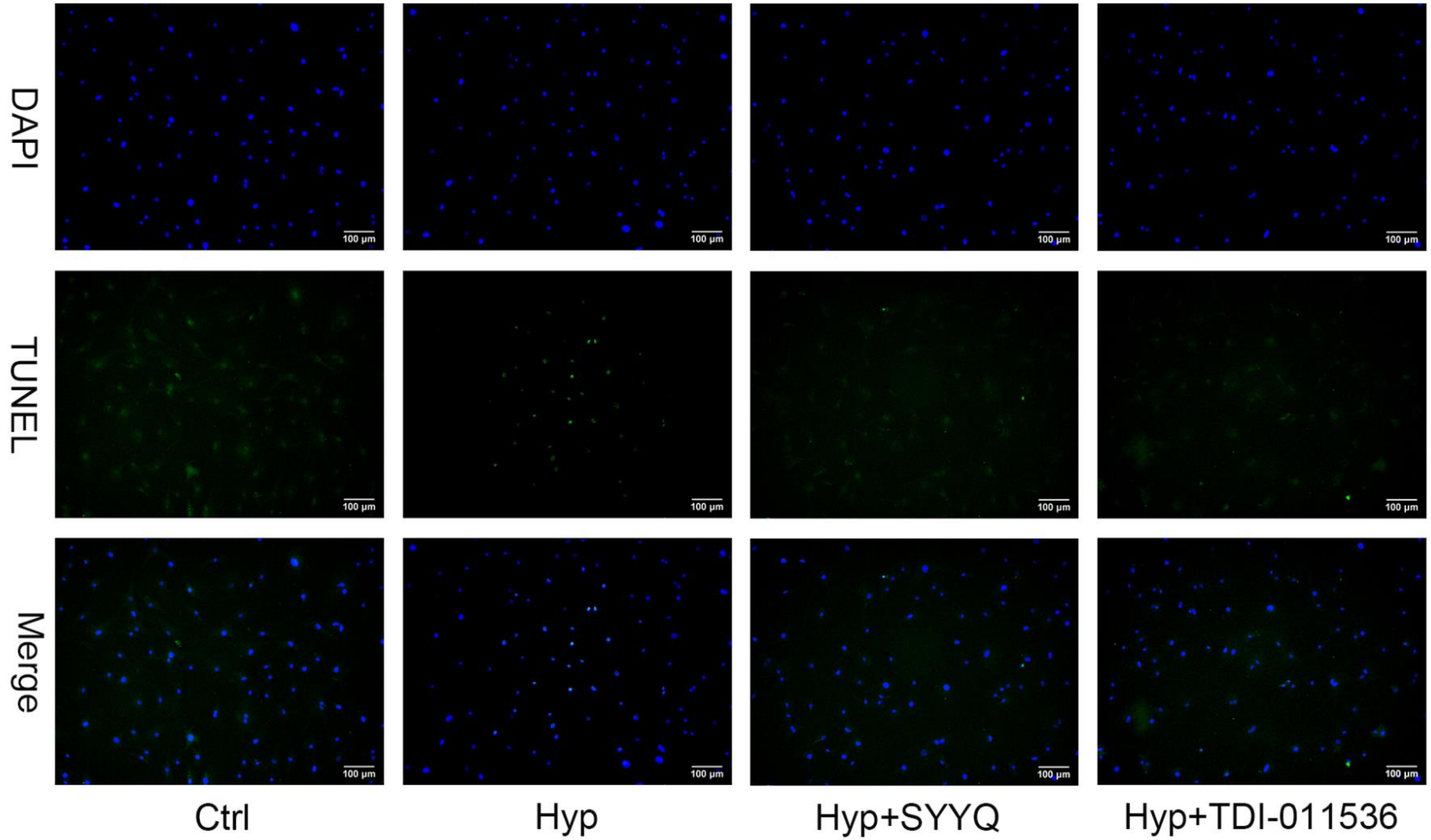

Supplementary figure F3 TUNEL assay to assess cell apoptosis rate *in vitro* (n=3)

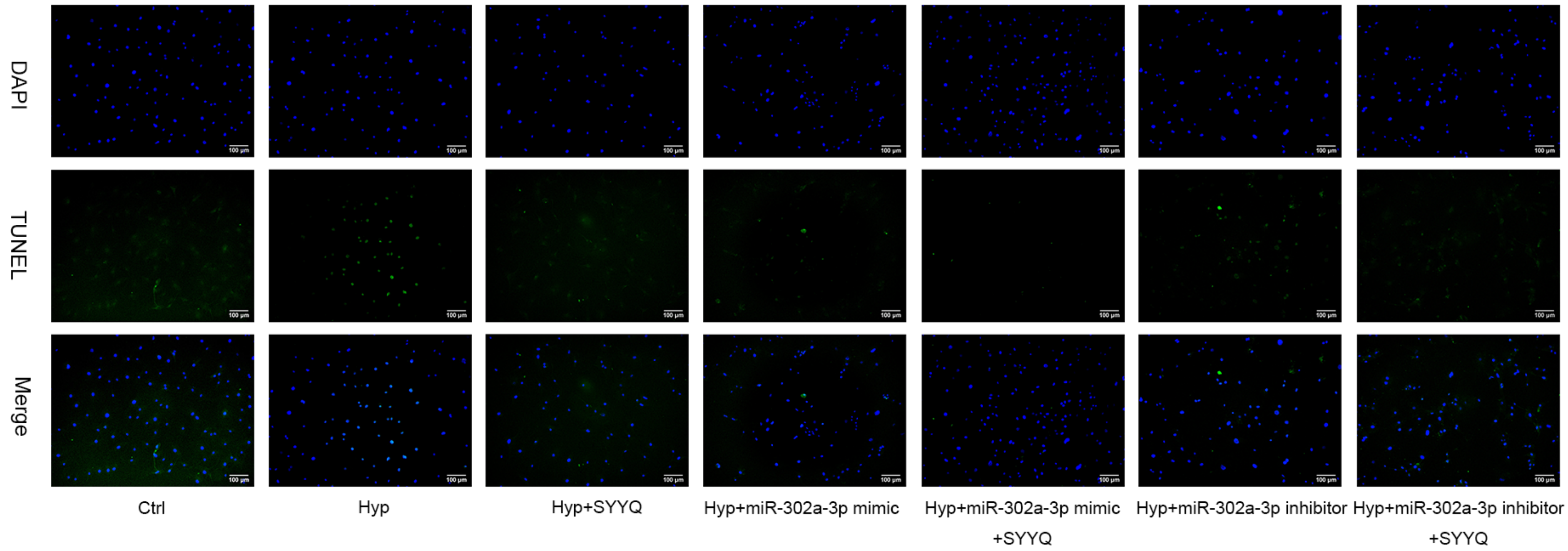

Supplementary figure F4 TUNEL assay to assess cell apoptosis rate *in vitro* (n=3)
